# Cerenkov radiation modulates the extracellular matrix for improved pancreatic cancer chemotherapy

**DOI:** 10.1016/j.celbio.2025.100221

**Published:** 2026-04-21

**Authors:** Weiwei Su, Han Wang, Shuai Zhao, Tao Wang, Jessica C. Hsu, Xiuru Ji, Shuping Li, Changjing Zuo, Weibo Cai, Dalong Ni

**Affiliations:** 1 Department of Radiology, Naval Medical Center, Shanghai, China; 2 Department of Nuclear Medicine, Changhai Hospital, Naval Medical University (Second Military Medical University), Shanghai, China; 3 Department of Orthopaedics, Shanghai Key Laboratory for Prevention and Treatment of Bone and Joint Diseases, Shanghai Institute of Traumatology and Orthopaedics, Ruijin Hospital, Shanghai Jiao Tong University School of Medicine, Shanghai, China; 4 Departments of Radiology and Medical Physics, University of Wisconsin-Madison, Madison, WI, USA; 5 Department of Biomaterials and Stem Cells, Suzhou Institute of Biomedical Engineering and Technology, Chinese Academy of Science, Suzhou, China; 6 These authors contributed equally; 7 Senior author; 8 Lead contact

## Abstract

Abundant cancer-associated fibroblasts (CAFs) in pancreatic tumors cause robust interstitial fibrosis and abnormal vascular structure, hindering the delivery and effectiveness of small-molecule therapeutics, leading to poor clinical outcomes. We developed a strategy to modulate the extracellular matrix (ECM) in pancreatic cancer to enhance chemotherapy through sequential administration of TiO_2_ nanoparticles (NPs), [^68^Ga] Ga-FAPI-04 ([^68^Ga]Ga-FAPI), and the chemotherapeutic drug tirapazamine (TPZ). The positron emission tomography tracer [^68^Ga]Ga-FAPI specifically targets CAFs and serves as an internal excitation source for Cerenkov radiation-mediated photodynamic therapy (CR-PDT). TiO_2_ NPs generate cytotoxic reactive oxygen species upon CR, destroying tumor cells and CAFs. As a result, CR-PDT reduces the dense stromal barrier by inhibiting the formation and secretion of fibrous collagen, improving the delivery of TPZ. Additionally, CR-PDT consumes oxygen throughout the process, intensifying tumor hypoxia, which further activates TPZ, a hypoxia-activated and bio-reductive prodrug. Our design of CR-PDT-mediated ECM modulation brings new ideas for the exploration and application of radiotracer-combined nanomedicine in cancer therapy, particularly for cancers with abundant ECM.

## In brief

The chemotherapeutic outcome of pancreatic cancer is limited by excessive extracellular matrix (ECM) with robust fibrosis, collapsed vessels and severe hypoxia. Su et al. applies Cerenkov radiation (CR) of clinic [^68^Ga]Ga-FAPI and nanosensitizers to modulate the ECM by damaging cancer associated fibroblasts, thus facilitating the delivery of chemotherapeutic drugs. Such design of CR-mediated ECM modulation for improved pancreatic cancer therapy will stimulate the application of radiotracer-combined nanomedicine for cancer treatments, particularly for cancers with abundant desmoplastic fibrous interstitium.

## Graphical Abstract

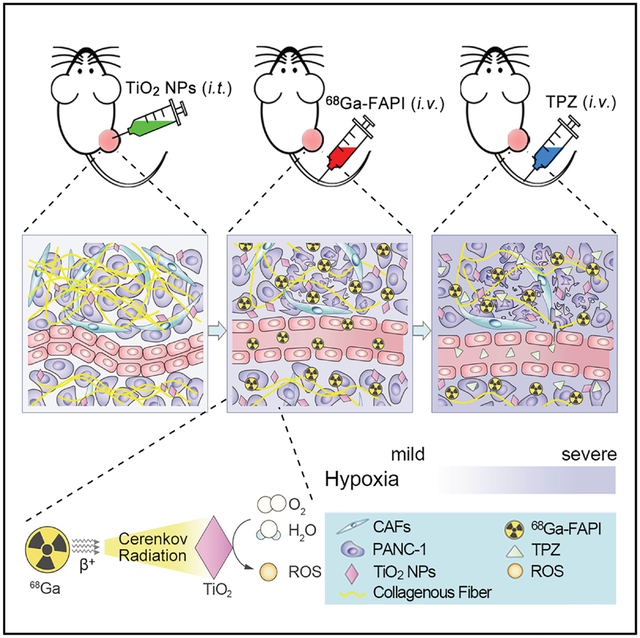

## INTRODUCTION

Pancreatic carcinomas are characterized by a robust fibroinflammatory or desmoplastic reaction, an excessive extracellular matrix (ECM), hypovascularity, and hypoxia, all of which contribute to their invasive nature and resistance to treatment.^1,2^ In the tumor microenvironment (TME), the fibrous collagen secreted by cancer-associated fibroblasts (CAFs)—the dominant cells in the tumor ECM—accounts for up to 90% of the tumor mass. This dense collagen deposition, along with elevated mechanical stress, causes approximately 75% of the blood vessels to compress or collapse.^3,4^ This collagen deposition and vascular collapse create a dense barrier that impedes the penetration and perfusion of chemotherapeutic drugs. The sparse and collapsed vasculature further exacerbates hypoxia in the TME, leading to drug resistance of the tumor.^5^ Hence, targeting CAFs to modulate the TME is a vital approach to improve the efficacy of chemotherapy.

Fibroblast activation protein (FAP), a type II transmembrane serine protease, is specifically and highly expressed by CAFs. With both dipeptidyl peptidase and endopeptidase activities, FAP cleaves collagen and fibronectin, thereby playing a critical role in the formation, secretion, and cross-linking of fibrous collagen, as well as stroma deposition and remodeling.^6,7^ Several studies have demonstrated the significant potential of coupling radionuclides (e.g., [^86^Y]Y, [^89^Zr]Zr, and [^177^Lu]Lu) to FAP inhibitors (e.g., FAPI-02, FAPI-04, and FAPI-46) for tumor-targeted imaging and therapy.^8–10^ The [^68^Ga]Ga-labeled FAPI ([^68^Ga]Ga-FAPI) has been clinically recognized as a tracer for CAF-targeting positron emission tomography/computed tomography (PET/CT) and widely used for targeted imaging of various tumors, including pancreatic cancer.^11,12^ However, [^68^Ga]Ga itself is a low-energy and short-distance β^+^ emitter, and [^68^Ga] Ga-FAPI is mainly used as a CAF-targeting PET/CT contrast agent in current clinical practice.

In the past decade, Cerenkov radiation-mediated photodynamic therapy (CR-PDT) has attracted considerable attention for cancer treatment. Traditional PDT typically uses ultraviolet (UV) and visible (vis) light to activate photosensitizers to generate reactive oxygen species (ROS). However, the limited tissue penetration of external light constrains the application of PDT for deep tumors. CR, emitted by multiple radionuclides (e.g., [^18^F]F, [^64^Cu]Cu, [^68^Ga]Ga), can act as an internal light source to excite photocatalytic materials and mediate CR-PDT. Among these radionuclides, [^68^Ga]Ga is particularly noteworthy because of its clinical availability, high yield of CR photons (33.9 per decay), and substantial electron energy (average 1,899 keV).^13–18^ Furthermore, as aforementioned, [^68^Ga]Ga-FAPI specifically targets tumor CAFs and thus may exert precise CR-PDT on CAFs. Furthermore, we speculate that the CAF-targeted CR-PDT may eliminate or inactivate CAFs, thereby inhibiting collagen production and cross-linking, degrading ECM components (e.g., hyaluronan), and decompressing blood vessels. Collectively, these effects may reduce the tumor stroma and significantly enhance the delivery of chemotherapeutic agents.

Traditional PDT, using organic photosensitizers, usually depends on oxygen content and is thus limited by the hypoxic TME. Recently, inorganic photosensitizers (e.g., TiO_2_) have attracted increasing attention. Our previous study, along with others, confirmed the favorable biocompatibility and catalytic performance of TiO_2_ nanoparticles (NPs).^17,19^ Upon CR, TiO_2_ generates electrons and holes. Holes oxidize water to generate hydroxyl radicals (a kind of ROS), which in turn damage cancer cells. This ROS-generation process is oxygen independent. Meanwhile, the CR-generated electrons transfer to other molecules, such as oxygen. Conduction band electrons can reduce residual oxygen to superoxide anion, thus exacerbating tumor hypoxia. Coincidently, tirapazamine (TPZ), a hypoxia-activated and bio-reductive prodrug, generates highly cytotoxic benzotriazinyl radicals under hypoxic conditions.^20^ TPZ acts as a substrate for intracellular reductase enzymes and obtains a single electron to form a free-radical intermediate. In the absence of oxygen in hypoxic cells, the free radicals cannot be oxidized back and instead remove hydrogen atoms of nearby macromolecules, like DNA, causing effects such as DNA double-strand breaks (DSBs), leading to chromosome aberrations and cell death.^21^ Several studies utilized TPZ as a radiosensitizer to address the insufficient therapeutic efficacy of radiotherapy resulting from tumor hypoxia, with research progressing to phase 3 clinical trials.^22,23^ Therefore, CR-PDT can operate in an oxygen-independent manner, and its intermediate side-reaction products can consume oxygen to intensify subsequent TPZ treatment.

Herein, we applied [^68^Ga]Ga-FAPI-04 (abbreviated as [^68^Ga] Ga-FAPI) and TiO_2_ NP-induced CR-PDT in combination with TPZ for pancreatic cancer therapy. First, TiO_2_ NPs were intratumorally injected into tumor tissue of mice. Next, [^68^Ga]Ga-FAPI was administered intravenously for three courses to target the tumor and initiate continuous CR-PDT, which destroyed cancer cells and CAFs, leaving a disrupted and loosen tumor stroma while aggravating hypoxic conditions. As a result, the hypoxia-activated TPZ effectively penetrated and perfused to the tumor bed, resulting in amplified anticancer effects of TPZ. Previous studies have attempted to regulate the TME to improve chemotherapy by interfering with intratumoral microbiomes,^24^ alleviating tumor hypoxia.^25^ Differently, this design of CR-PDT-mediated ECM modulation for TME regulation, integrated with chemotherapy, may inspire further exploration of radiotracercombined nanomedicine treatments for cancer, particularly for tumors with abundant desmoplastic fibrous interstitium.

## RESULTS AND DISCUSSION

### Bioinformatic analysis of FAP in patients with PAAD

**Figure 1.**
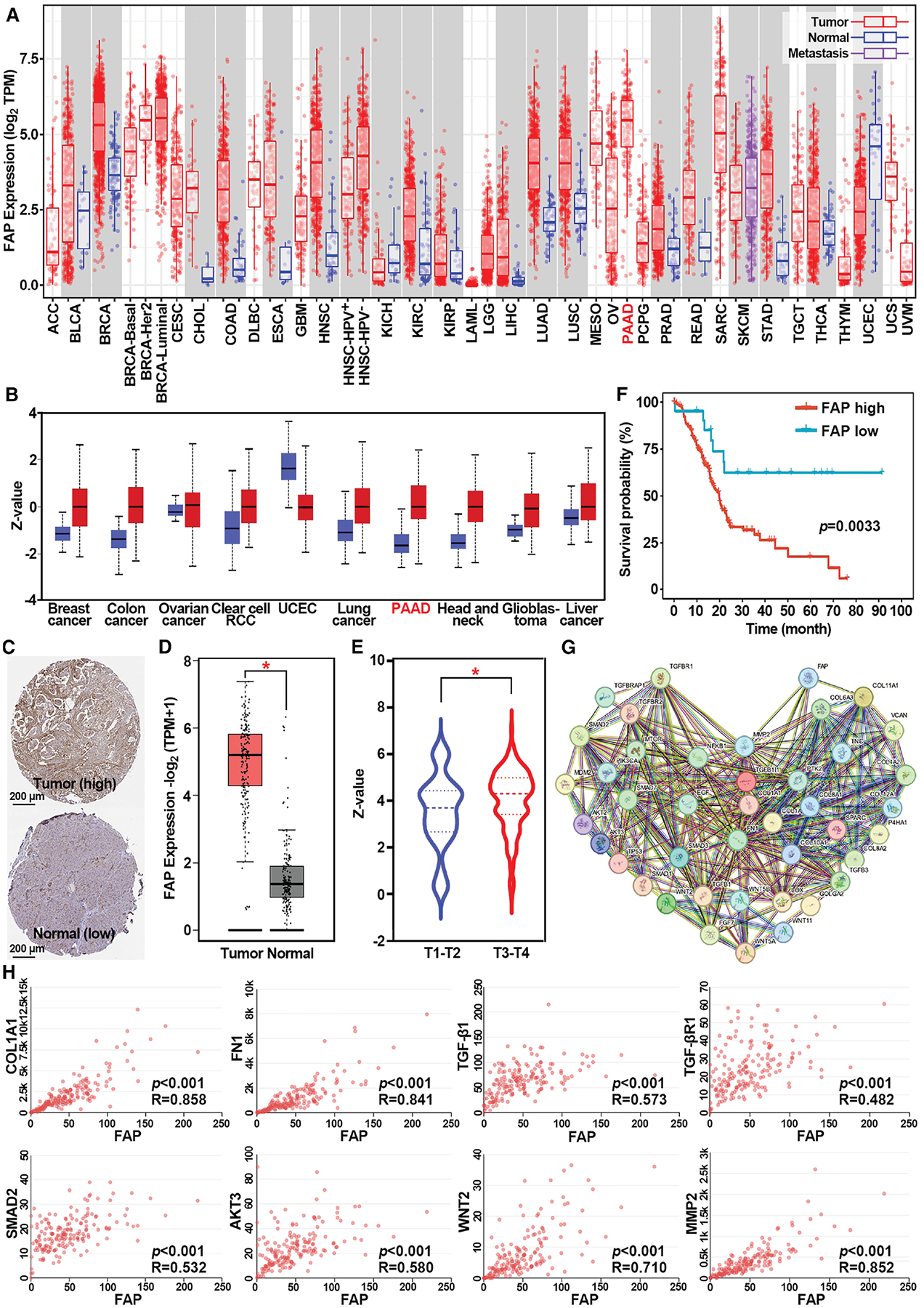
Bioinformatic analysis of FAP in patients with PAAD (A) Transcript level of FAP in various tumor types of human. (B) Expression of FAP protein in various tumor types of human. (C) Evaluation of FAP expression in PAAD and normal pancreatic tissue by H&E staining. (D) Boxplots of mRNA expression of FAP in PAAD and normal pancreatic tissue (log_2_ |fold change [FC]| > 1, *p < 0.01). (E) Expression of FAP in PAAD at different pathological T stages. (F) Relationship of FAP expression level with the overall survival of patients with PAAD. (G) The protein-protein interaction (PPI) network of FAP with proteins related to tumor fibrosis. (H) Scatterplots for the correlation between FAP and proteins related to tumor fibrosis in PAAD.

Bioinformatic analysis for the CAF biomarker FAP was conducted using clinical data of patients with pancreatic adenocarcinoma (PAAD). Analysis of The Cancer Genome Atlas and Human Protein Atlas database revealed that FAP was highly expressed at both protein (Figure 1A) and RNA (Figure 1B) levels in multiple tumor types, including PAAD, in which the FAP expression was significantly higher in tumor tissue than adjacent normal pancreatic tissue (Figures 1C and 1D). What’s more, FAP expression showed an increased trend in PAAD at an advanced T stage (Figure 1E) and a higher tumor grade (Figure S1). Prognostic analysis indicated that PAAD patients with higher FAP level showed significantly shorter overall survival (Figure 1F). These results indicated the potential link between FAP and the progression and prognosis of PAAD, which was consistent with previous reports.^26,27^

Further, multiple proteins were found to be correlated with FAP, among which we paid attention to collagen I (COL1A1), fibronectin 1 (FN1), matrix metalloproteinase 2 (MMP2), small mother against decapentaplegic 2 (SMAD2), recombinant wingless-type mouse mammary tumor virus integration site family member 2 (WNT2), transforming growth factor beta 1 (TGF-β1), TGF-β receptor I (TGF-βR1), and protein kinase B (AKT). These proteins were related to tumor fibrosis by being involved in the formation of stroma collagen or fiber or by regulating related enzymes or signaling pathways, and they were therefore selected for subsequent analysis. Protein-protein interaction network displayed interactions between FAP and these proteins in PAAD (Figure 1G), and scatterplots displayed a positive correlation between FAP and each of these proteins of interest (Figure 1H). Together, these results derived from clinical data of PAAD patients indicate the potential role of FAP (and thus CAFs) in tumor fibrosis, and they provide a theoretical foundation for treatment of pancreatic tumors.

### Synthesis and characterization of TiO_2_ NPs

**Figure 2.**
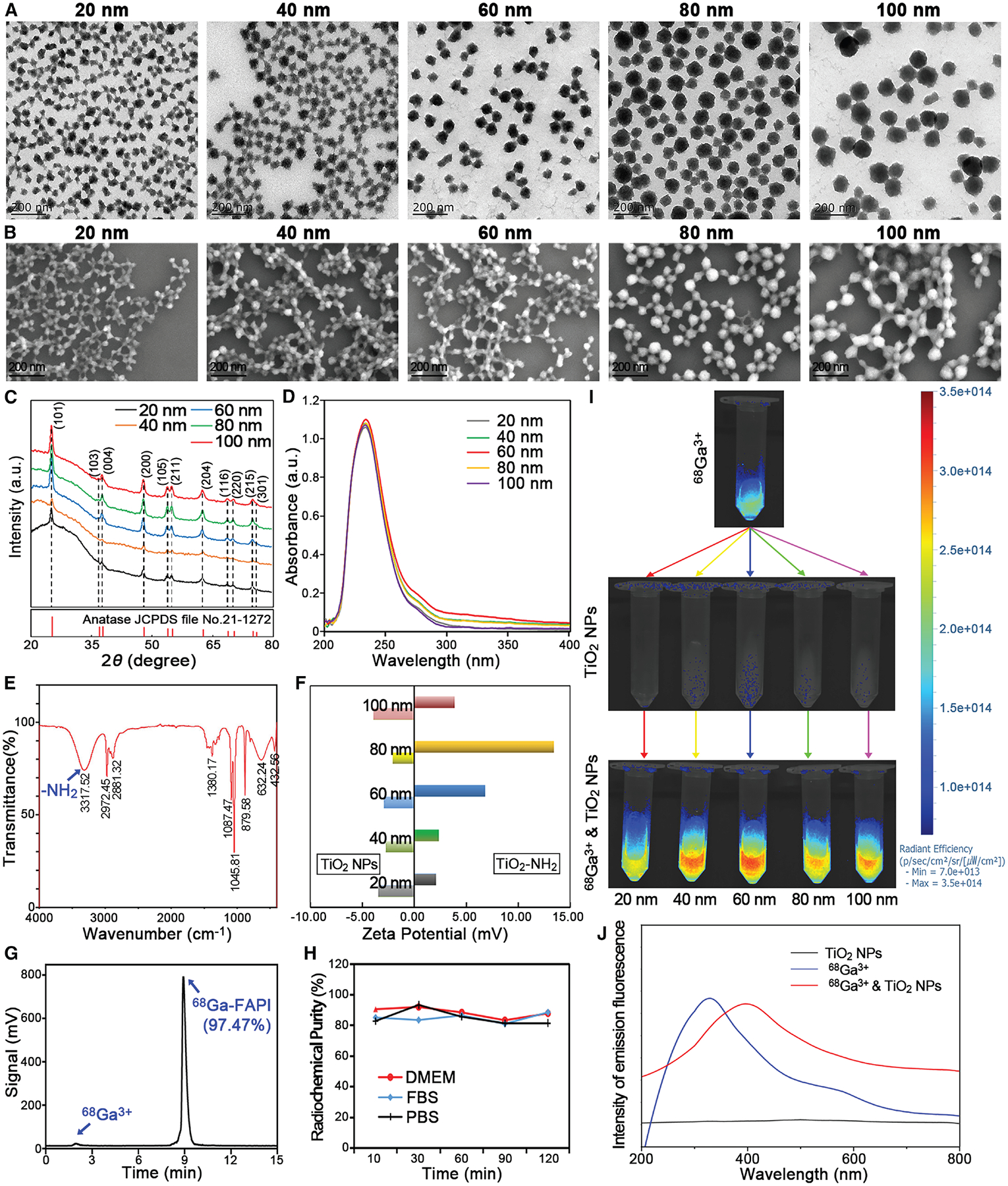
Synthesis, characterization of materials, and mechanism verification in aqueous solution (A) TEM images of TiO_2_ NPs of different sizes. (B) SEM images of TiO_2_ NPs of different sizes. (C) XRD patterns of TiO_2_ NPs of different sizes. (D) UV-vis spectra of TiO_2_ NPs of different sizes. (E) FTIR spectra of TiO_2_-NH_2._ (F) Zeta potentials of TiO_2_ NPs and TiO_2_-NH_2_. (G) Radio labeling rate of [^68^Ga]Ga-FAPI determined by HPLC. (H) *In vitro* stability test of [^68^Ga]Ga-FAPI. (I) CRET analysis from [^68^Ga]Ga to TiO_2_ NPs using an IVIS instrument. (J) Emission spectra of ^68^Ga, TiO_2_ NPs, and ^68^Ga and TiO_2_ NPs.

First, TiO_2_ NPs of five different sizes (mean ± SD)—20 nm (23.5 ± 4.3 nm), 40 nm (38.4 ± 6.1 nm), 60 nm (57.5 ± 5.5 nm), 80 nm (78.7 ± 7.0 nm), and 100 nm (99.4 ± 9.5 nm)—were prepared, based on the consideration that the catalytic activity of TiO_2_ NPs may be influenced by particle size.^28–30^ Transmission electron microscopy (TEM) images confirmed that TiO_2_ NPs of all five sizes were uniform with a well-defined spherical shape (Figure 2A). Scanning electron microscopy (SEM) images demonstrated good dispersibility and structural integrity of all TiO_2_ NPs (Figure 2B). The TiO_2_ NP solution was tested using dynamic light scattering (DLS), and the hydrodynamic radii of the five groups of TiO_2_ NPs were 36.7, 63.8, 92.5, 129.1, and 140.0 nm, respectively (Figure S2). After incubating TiO_2_ NPs in phosphate buffer solution (PBS) or fetal bovine serum (FBS) for 24 h and 7 days, no change in the hydrodynamic radii was observed, indicating good physiological stability of the TiO_2_ NPs (Figure S3). X-ray diffraction (XRD) patterns, with prominent diffraction peaks at a 2θ angle of 25.3°, corresponding to the anatase (101) crystal face, confirmed the successful synthesis of TiO_2_ NPs (Figure 2C). UV-vis spectra of the five types of TiO_2_ NPs showed similar absorption peaks at 233–234 nm, with the 60-nm TiO_2_ NPs showing a slightly higher absorption peak value, compared with the others (Figure 2D). Furthermore, the TiO_2_ NPs were modified with NH_2_ groups (TiO_2_-NH_2_) to facilitate the conjugation of indocyanine green (TiO_2_-ICG), which allows for monitoring intratumoral localization and retention via fluorescence imaging. The absorption peak of NH_2_ on Fourier transform infrared spectroscopy (FTIR) spectra (Figure 2E) and the increased hydrodynamic radii (57.1, 89.7, 106.6, 146.9, and 167.4 nm, respectively; Figure S4) and zeta potential (Figure 2F) indicated successful NH_2_ modification. Element analysis by using energy dispersive spectroscopy (EDS) identified the presence of Ti, O, and N in TiO_2_ NPs (Figure S5).

The [^68^Ga]Ga-FAPI tracer was prepared using a traditional method.^31^ The initial labeling efficiency recorded using high-performance liquid chromatography (HPLC) was as high as 97.47% (Figures 2G and S6) and remained above 80% after 2 h of incubation in 10% FBS, Dulbecco’s Modified Eagle Medium (DMEM), and PBS, signifying its favorable *in vitro* stability (Figure 2H).

To investigate and compare the CR energy transfer (CRET) from [^68^Ga]Ga to TiO_2_ NPs, an *in vivo* imaging system (IVIS) instrument was used. The fluorescence signal of 60-nm TiO_2_ NPs was brightest after a 10-min incubation with [^68^Ga]Ga (Figure 2I). Furthermore, the emission spectra of TiO_2_ NPs, ^68^Ga, and the mixture of TiO_2_ NPs and ^68^Ga were examined. A red shift in the emission spectra from 280 to 350 nm was observed upon mixing ^68^Ga with TiO_2_ NPs, compared with ^68^Ga alone, providing more evidence for the CR energy transformation from [^68^Ga]Ga to TiO_2_ NPs (Figure 2J). Hence, TiO_2_ NPs with a size of 60 nm were selected for subsequent experiments.

### Intracellular distribution and intratumoral accumulation of TiO_2_ NPs and [^68^Ga]Ga-FAPI

**Figure 3.**
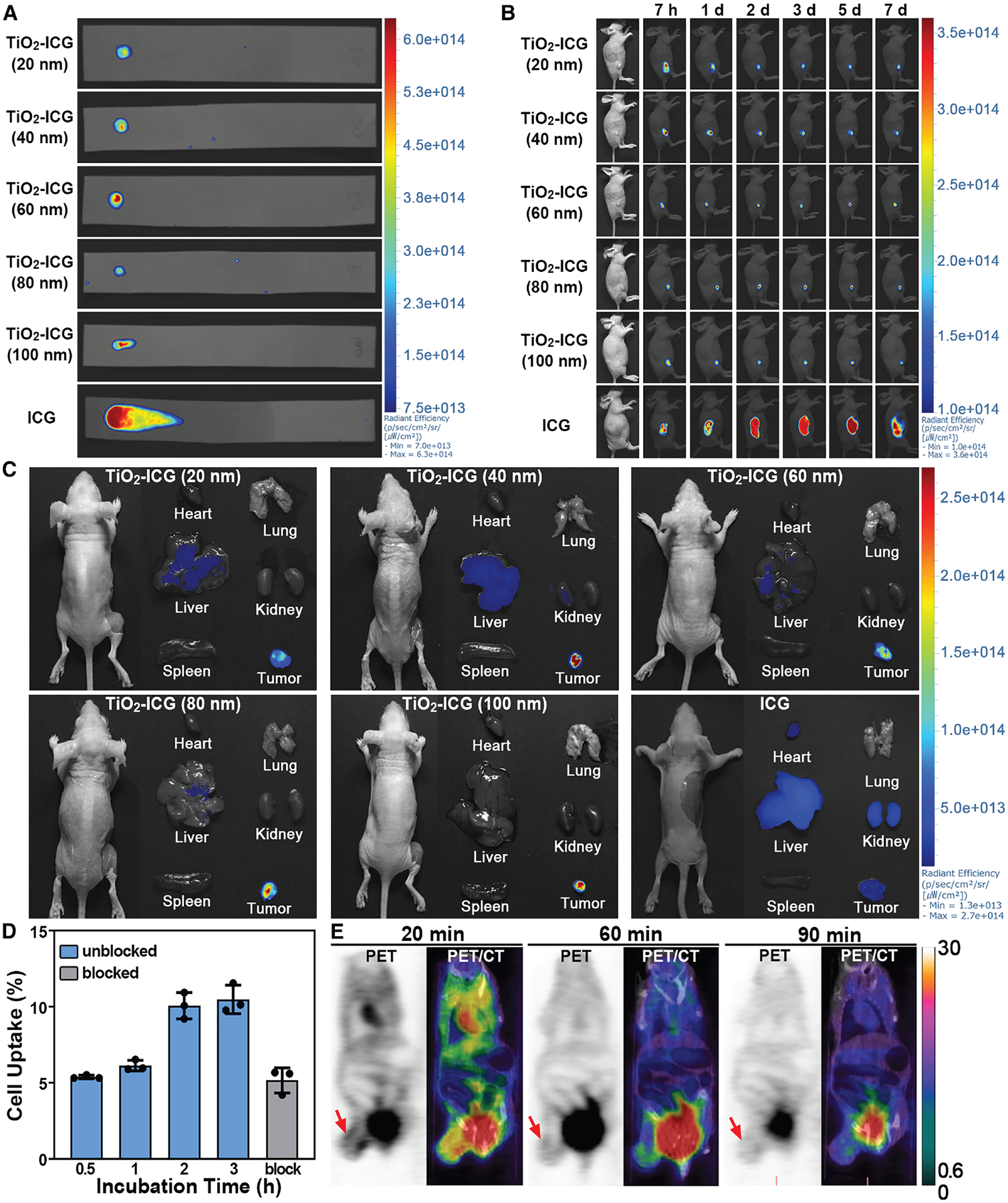
Evaluation of the intratumoral retention of TiO_2_ NPs and the tumor targeting and intratumoral retention of [^68^Ga]Ga-FAPI (A) Labeling efficiency of TiO_2_-ICG compared with the ICG control, using a chromatographic test strip. (B) *In vivo* fluorescence imaging of TiO_2_-ICG or ICG at different time points up to 7 days after intratumoral injection. (C) *Ex vivo* fluorescence imaging of tumors and major organs 7 days post-injection of TiO_2_-ICG or ICG. (D) CAF-specific cellular competitive binding assay for [^68^Ga]Ga-FAPI (*n* = 3, mean ± SD). (E) PET/CT imaging assessing the tumor targeting and biodistribution of [^68^Ga]Ga-FAPI.

Intracellular uptake and intratumoral accumulation of TiO_2_ NPs and [^68^Ga]Ga-FAPI are the preconditions for efficient therapeutic outcome through CR-PDT. We first investigated the cellular uptake of TiO_2_ NPs in pancreatic carcinoma cell line (PANC-1) and CAFs under confocal laser scanning microscopy (CLSM) by labeling TiO_2_ NPs with fluorescein isothiocyanate (FITC). We found that most FITC-NPs were endocytosed and mainly located in the cytoplasm of both PANC-1 cells and CAFs after 5 h of incubation (Figure S7). To better replicate the real TME, a subcutaneous pancreatic cancer model was then established in BALB/c nude mice by co-injecting equal numbers of PANC-1 cells and CAFs into the right posterior limb of each mouse.^32^ Since previous studies indicated that the accumulation of NPs in and their penetration into the tumor were correlated with particle size,^33,34^ we investigated the intratumoral retention of all five sizes of TiO_2_ NPs after intratumoral injection. In detail, ICG-labeled TiO_2_ NPs (TiO_2_-ICG) were synthesized with a high labeling efficiency (Figure 3A). ICG or TiO_2_-ICG with TiO_2_ NPs of various sizes were intratumorally injected, and their distribution was monitored using the IVIS instrument. *In vivo* imaging showed a continuously strong fluorescence signal at the tumor site over different time points within 7 days following TiO_2_-ICG injection (Figures 3B and S8). *Ex vivo* biodistribution analysis conducted on the 7^th^ day post-injection exhibited the strongest fluorescence signal in tumors but only a faint signal in liver, particularly in mice injected with 60-nm TiO_2_ NPs (Figure 3C). This indicates that TiO_2_ NPs with a size of 60 nm or larger were more superior in long-term deposition and retention at the tumor site. Next, TiO_2_ NPs (60 nm) were labeled with cyanine 5 amine (TiO_2_-Cy5) and administered to tumor-bearing mice by using the multi-point intratumoral injection method. At 1 day post-injection, tumors were dissected, stained with DAPI (to stain the cell nucleus) and CK19 (an epithelial-derived marker), and imaged. As shown in Figure S9, abundant Cy5-TiO_2_ NPs were diffusely distributed throughout the tumor tissue, ensuring broad therapeutic efficacy. On the basis of these above results, we selected TiO_2_ NPs with a size of 60 nm for subsequent experiments.

To evaluate the targeting ability of [^68^Ga]Ga-FAPI, a cellular competitive binding assay was first conducted in CAFs. The cellular uptake of FAPI by CAFs increased from 5.38% to 10.49% as the incubation time extended from 0.5 to 3 h (Figure 3D). By contrast, CAFs pre-treated with an excess dose (250 times) of unlabeled FAPI-04, used as a competitive agent, showed a lower uptake of only 5.16% after 3 h of incubation. Next, [^68^Ga]Ga-FAPI was intravenously injected into mice, and its *in vivo* biodistribution was dynamically monitored using PET/CT scanning. As shown in Figure 3E, at 20 min post-injection, [^68^Ga]Ga-FAPI rapidly and selectively targeted the tumor over other organs, achieving a maximum standardized uptake value (SUVmax) of tumor-to-background ratio of the liver (TBRliver) of 1.14. With the tracer gradually cleared from the liver over time, the calculated TBRliver value increased from 1.14 at 20 min post-injection to 1.53 at 90 min post-injection (Figure S10). Thus, [^68^Ga]Ga-FAPI demonstrates excellent tumor specificity and retention, ensuring effective CR-PDT function before [^68^Ga]Ga nearly decays out.

### *In vitro* effect of CR-PDT&TPZ on PANC-1 cells

**Figure 4.**
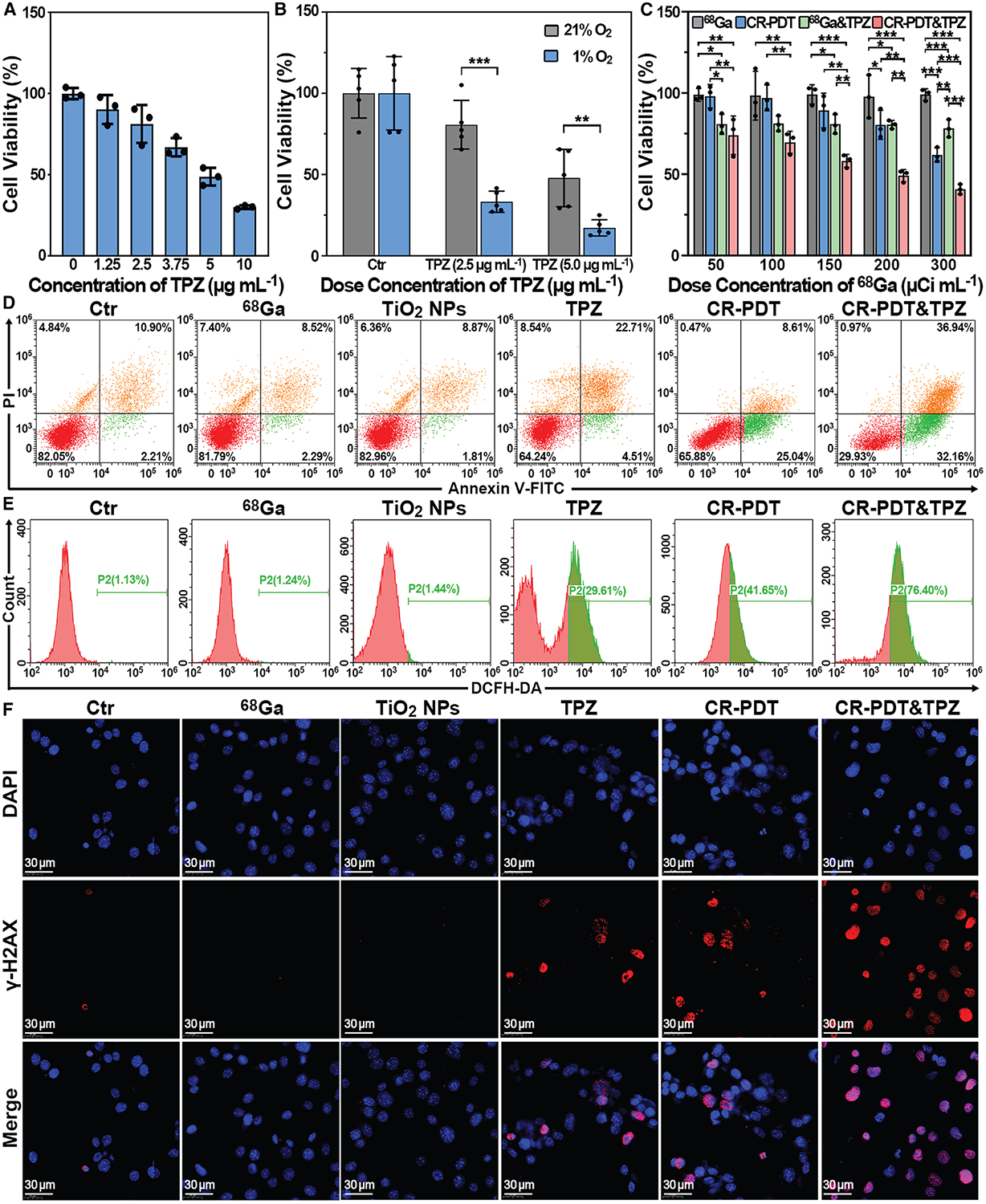
*In vitro* cytotoxicity assessment of CR-PDT&TPZ in PANC-1 cells (A) CCK-8 assay evaluation of the dose-dependent cytotoxicity of TPZ in PANC-1 cells (*n* = 3, mean ± SD). (B) CCK-8 assay evaluation of the hypoxia-dependent cytotoxicity of TPZ in PANC-1 cells (*n* = 5, mean ± SD, **p < 0.01, ***p < 0.001, one-way analysis of variance [ANOVA] with least significant difference [LSD] t test). (C) CCK-8 assay to determine the optimal dosage of CR-PDT and TPZ in PANC-1 cells with varying [^68^Ga]Ga concentrations (*n* = 3, mean ± SD, *p < 0.05, **p < 0.01, ***p < 0.001, one-way ANOVA with LSD t test). (D) Flow cytometry analysis of cell apoptosis in different groups. (E) Flow cytometry measurement of ROS production in different groups. (F) Immunofluorescence assay analyzing DNA DSBs in different groups.

To evaluate the treatment effectiveness of CR-PDT combined with TPZ (CR-PDT&TPZ), a preliminary experiment was conducted in PANC-1 tumor cells. As shown in Figure S11, TiO_2_ NPs alone exhibited a negligible detrimental impact on cell viability even at a high concentration up to 1,200 μg mL^−1^, indicating that TiO_2_ NPs were safe within a certain concentration range. Under normoxic conditions (O_2_ concentration 21%), cell viability decreased with increasing TPZ concentration, demonstrating the dose-dependent cytotoxicity of TPZ (Figure 4A). Cells treated with TPZ (2.5 μg mL^−1^) under hypoxic conditions (O_2_ concentration 1%) exhibited significantly lower viability (33.27%), compared with those treated under normoxic conditions (80.65%) (Figure 4B). This confirmed the hypoxia-activated and bio-reductive nature of TPZ.

To more closely explore the mechanism of the enhanced tumor killing by TPZ in hypoxic conditions, ROS production and DNA damage were evaluated in cells treated with TPZ under normoxic and hypoxic conditions. As shown in Figures S12 and S13, TPZ induced more ROS generation (61.39%) and more severe DNA DSBs (γ-H_2_AX positive rate 90.84%) in cells under a hypoxic environment than in those under a normoxic environment (ROS 33.30%), which was consistent with a previous study.^20^

Next, the optimal dosage of [^68^Ga]Ga was explored. As shown in Figure 4C, [^68^Ga]Ga alone caused negligible cell death in PANC-1 tumor cells even at a concentration of 11.1 MBq mL^−1^ (300 μCi mL^−1^), while CR-PDT resulted in increasing cell death in a dose-dependent manner. At a [^68^Ga]Ga concentration of 7.4 MBq mL^−1^ (200 μCi mL^−1^), CR-PDT&TPZ led to approximately 50.90% cell death, which was significantly higher than that of CR-PDT alone (19.63%) or TPZ alone (19.36%). The viability of cells treated with [^68^Ga]Ga&TPZ was similar to that of cells treated with TPZ alone (80.59% vs. 80.64%), which further supported the negligible detrimental effect of [^68^Ga]Ga, and only when it reacted with the TiO_2_ NPs, the cell killing effect of CR-PDT can be exerted. Therefore, we selected a dosage of 7.4 MBq mL^−1^ (200 μCi mL^−1^) of [^68^Ga] Ga, 150 μg mL^−1^ of TiO_2_ NPs, and 2.5 μg mL^−1^ of TPZ for subsequent experiments.

The cytotoxic effects of CR-PDT and TPZ are primarily due to the production of ROS, which can directly induce DNA DSBs and subsequent apoptosis.^35^ We thus evaluated cell apoptosis using annexin V-FITC/propidium iodide (PI) staining and flow cytometry. As shown in Figures 4D and S14, the CR-PDT&TPZ group triggered the most severe cell apoptosis (apoptosis rate, 59.57%), which was 1.90, 2.84, and 4.89 times that of the CR-PDT, TPZ, and control groups, respectively. Furthermore, intracellular ROS was measured using the 2′,7′-dichlorodihydrofluorescein diacetate (DCFH-DA) probe. As illustrated in Figures 4E and S15, the amount of ROS in the CR-PDT&TPZ group increased substantially after 24 h of treatment and was 1.71, 3.25, and 65.25 times that of the CR-PDT, TPZ, and control groups, respectively. Further, DNA DSBs were examined via immunofluorescence for γ-H_2_AX. As shown in Figures 4F and S16, PANC-1 tumor cells treated with CR-PDT&TPZ exhibited the most DNA DSBs, compared with those treated with CR-PDT or TPZ alone, with levels 1.60 and 1.99 times that of CR-PDT and TPZ groups, respectively. These data confirmed that the combined therapy with CR-PDT&TPZ had greater killing effects on tumor cells, compared with single treatments.

The above data indicated that CR-PDT resulted in intensification of hypoxia to amplify the effects of TPZ. To obtain direct visual evidence, the intracellular oxygen condition in cells treated with CR-PDT was monitored using the hypoxia-sensitive fluorescent probe BBoxiProbe O91. This probe contains a nitro-aromatic group that is selectively reduced by intracellular nitro-reductase (NTR) under hypoxic conditions, generating a green fluorescence signal. As shown in Figures S17 and S18, cells in control, [^68^Ga]Ga, and TiO_2_ NPs groups exhibited minimal fluorescence, whereas CR-PDT-treated cells displayed markedly intensified signals, confirming pronounced oxygen depletion. Further, to validate hypoxia induction, the expression of hypoxia-inducible factor-1α (HIF-1α) was examined, which was a master regulator of the cellular responses to low oxygen. Immunofluorescence staining revealed significantly elevated HIF-1α levels in CR-PDT-treated cells, compared with other groups, indicating induced cellular hypoxia (Figures S17 and S18). All these results collectively demonstrated CR-PDT-mediated hypoxia amplification, providing a mechanism for its enhancement of TPZ cytotoxicity in tumor therapy.

### *In vivo* performance of CR-PDT&TPZ

**Figure 5.**
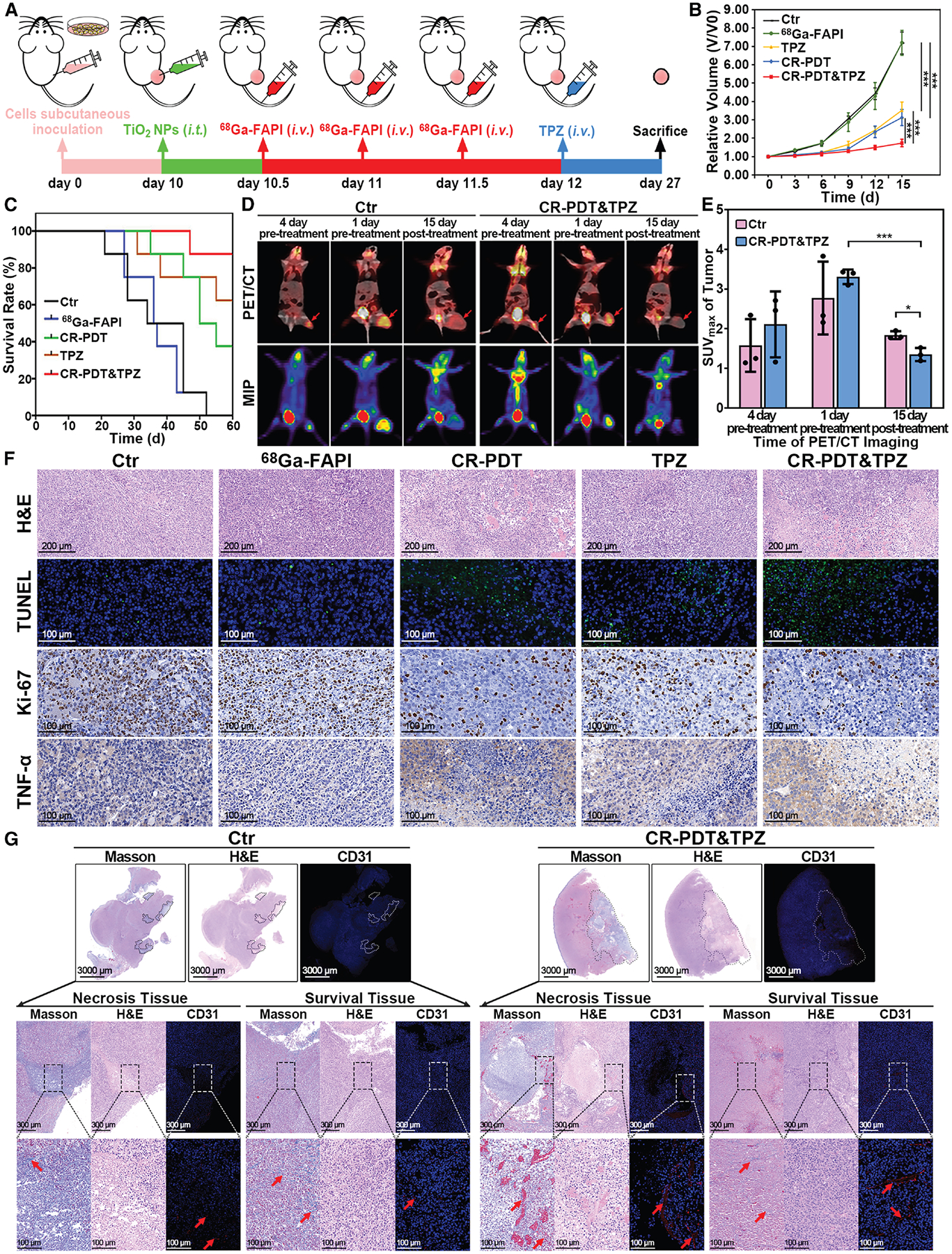
*In vivo* efficacy of CR-PDT&TPZ in tumor-bearing mice (A) Schematic diagram of the treatment regimen of CR-PDT&TPZ. (B) Tumor growth curves during the 15-day treatment period. Relative tumor volume (V V_0_^−1^) was determined by normalizing the tumor volume (V) to the initial value (V_0_) (*n* = 5, mean ± SD, ***p < 0.001, one-way analysis of variance (ANOVA) with LSD t test). (C) Kaplan-Meier survival curves of mice in the indicated groups during the 60-day observation period (*n* = 8). (D) [^18^F]F-FDG PET/CT imaging accessing tumor growth and glucose metabolism in different groups (top: PET/CT images, bottom: maximum intensity projection [MIP] images). (E) Semi-quantitative analysis based on SUVmax in [^18^F]F-FDG PET/CT (*n* = 3, mean ± SD, *p < 0.05, ***p < 0.001, Mann-Whitney U test). (F) H&E, TUNEL, and immunohistochemistry images of Ki-67 and TNF-α in tumor specimens collected on day 15 after treatment (original magnification: 200× for H&E images and 400× for other images). (G) Masson staining, immunofluorescence staining of CD31, and H&E staining for evaluation of collagen fibers, vessels, and tumor necrosis in the CR-PDT&TPZ and control groups (original magnification: top, 4×; middle, 50×; bottom, 200×).

To evaluate the *in vivo* treatment efficacy of CR-PDT&TPZ, tumor-bearing mice were generated and randomly divided into five groups: control, [^68^Ga]Ga-FAPI, CR-PDT, TPZ, and CR-PDT&TPZ. Considering the short half-life and limited CR energy of [^68^Ga]Ga, one course of CR-PDT may not be sufficient to yield enough ROS to induce thorough CAF damage, degrade the ECM, and effectively contribute to the import of TPZ. Therefore, we improved the administration manner as follows: one course of TiO_2_ NP implantation was followed by three intravenous injections of [^68^Ga]Ga-FAPI. Controllable administration of [^68^Ga]Ga-FAPI can repeatedly excite TiO_2_ NPs, produce abundant ROS, and enable successive attacks on CAFs, thus realizing efficient CR-PDT. The treatment protocol for the CR-PDT&TPZ group is illustrated in Figure 5A. Mice received an intratumoral injection of TiO_2_ NPs (700 μg), followed by three intravenous injections of [^68^Ga]Ga-FAPI (27.9 MBq [755 μCi] per mouse) at 12-h intervals (approximately 10 half-life periods of [^68^Ga]Ga). Then, 12 h after the final [^68^Ga]Ga-FAPI injection, TPZ (50 μg per mouse) was administered intravenously.

After 15 days, tumors in the control and [^68^Ga]Ga-FAPI groups progressed to 7.18- and 7.20-fold of their initial volumes, respectively (Figures 5B and S19). In contrast, tumors treated with CR-PDT, TPZ, or CR-PDT&TPZ only grew to 3.12-, 3.52-, and 1.73-fold of their initial sizes, respectively. During the 60-day observation period, the survival time of mice in the CR-PDT&TPZ group (mean survival time, 58.4 days) was significantly longer, compared with other groups, with only one mouse death on day 47 post-injection (Table S1). Conversely, all mice in the control (median survival time, 34.0 days) and [^68^Ga]Ga-FAPI groups (median survival time, 36.0 days) quickly died out. In contrast, three mice treated with CR-PDT (median survival time, 50.0 days) and five mice treated with TPZ (mean survival time, 53.0 days) survived until day 60 post-injection (Figure 5C; Table S1). In group comparisons, mice in the CR-PDT group showed significantly prolonged survival, compared with those in the control (χ^2^ = 6.997, *p* = 0.008) and [^68^Ga]Ga-FAPI groups (χ^2^ = 7.392, *p* = 0.007). While mice in the CR-PDT&TPZ group exhibited longer survival than those in the CR-PDT (χ^2^ = 3.755, *p* = 0.053) and TPZ (χ^2^ = 1.317, *p* = 0.251) groups, these differences did not reach statistical significance (Table S2). The lack of significance may be influenced by the limited observation period and needs to be explored in future studies. Additionally, all the mice maintained relatively stable body weights throughout the observation period, with no signs of cachexia (Figure S20). On day 15 post-injection, [^18^F]F-FDG PET/CT imaging showed significant inhibition of tumor growth in the CR-PDT&TPZ group, with the level of glucose metabolism decreased by 26.49%, as quantified by SUVmax, compared with that in the control group (Figures 5D and 5E).

After the treatment regimen was completed, tumors were collected for pathological and histopathological examinations, including hematoxylin and eosin (H&E) staining, terminal deoxy-nucleotidyl transferase dUTP nick end labeling (TUNEL) staining, and immunohistochemical staining for tumor necrosis factor alpha (TNF-α), pro-apoptotic proteins (Bax, caspase-3), and a cell proliferation factor (Ki-67) (Figures 5F and S21). As shown in Figure S22, the quantitative results demonstrated that CR-PDT&TPZ represents a highly efficient anticancer treatment approach, with most obvious tumor necrosis, apoptosis, and proliferation inhibition.

Additionally, in the control group, Masson’s trichrome staining revealed a mass of tangled collagen fibrils in the tumor, especially in the necrotic area rather than the surviving area. CD31^+^ vessels were extremely sparse, with collapsed lumens and twisted shapes (Figure 5G), which is consistent with the manifestation of tumor necrosis caused by poor blood supply. Conversely, in the CR-PDT&TPZ group, the necrotic tumor tissue showed relatively less collagenous fiber, with abundant vessels with dilated lumens and increased vascular permeability. The decompressed and expanded vasculature resulting from fibrous degradation did not appear to improve the tumor’s blood supply but rather killed more cancer cells. This effect may be attributed to the increased delivery and penetration of TPZ across the tumor bed. H&E analysis for the major organs demonstrated the biosafety of all treatments (Figure S23). These findings primarily support our hypothesis regarding ECM modulation as a strategy to enhance tumor treatment efficacy.

### Tumor ECM modulating and hypoxia intensification

**Figure 6.**
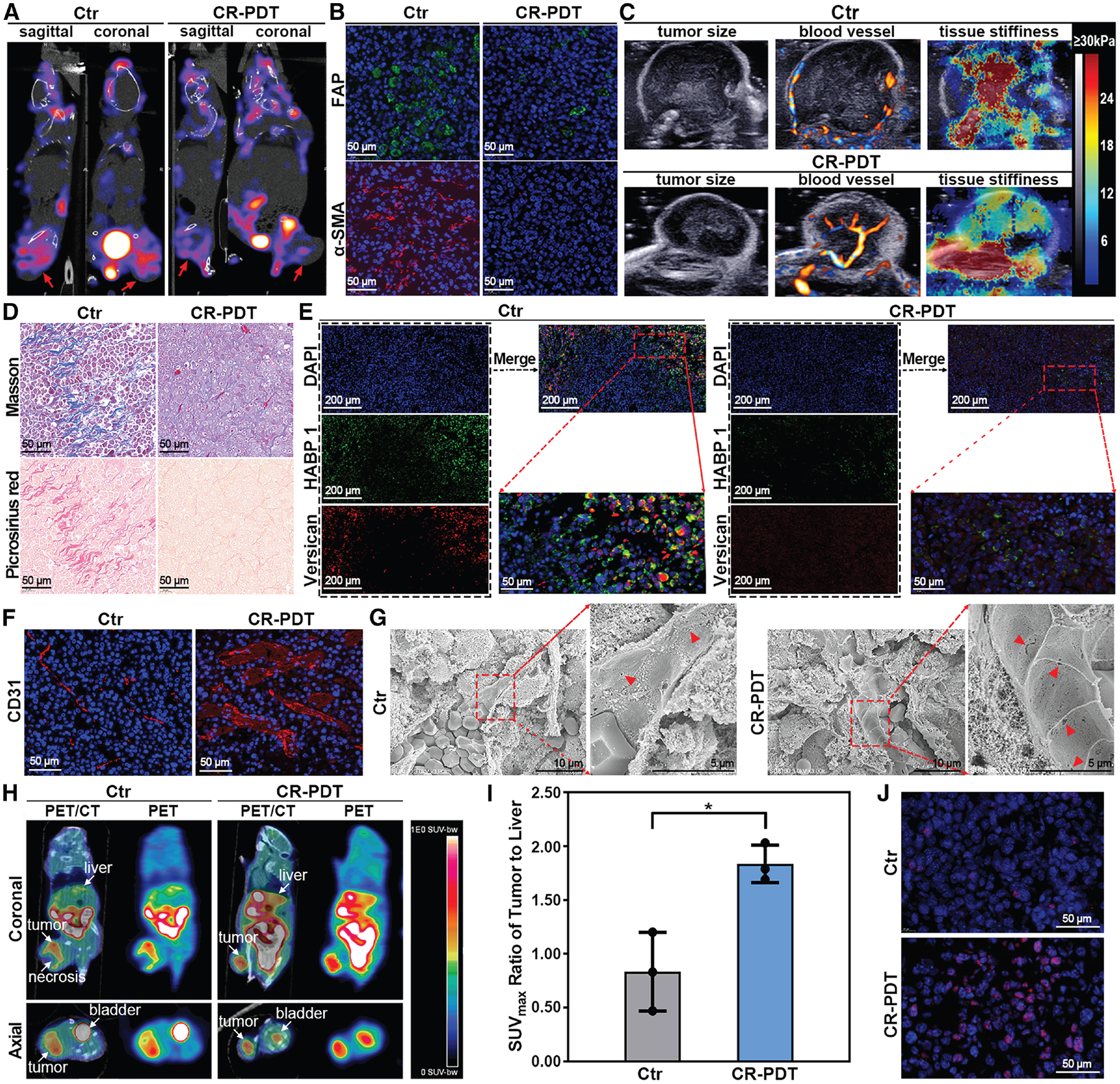
The underlying treatment mechanism of CR-PDT (A) [^99m^Tc]Tc-FAPI SPECT/CT imaging of mice in the CR-PDT and control groups. (B) Immunofluorescence staining of the expression of FAP and α-SMA in tumors of the CR-PDT and control groups (original magnification: 400×). (C) Ultrasonic elastography detection of the stiffness and vessels of tumor in the CR-PDT and control groups. (D) Masson’s trichrome stain and PSR stain of tumor tissue in the CR-PDT and control groups (original magnification: 400×). (E) Immunofluorescence double staining for HABP1 and versican in tumors of the CR-PDT and control groups (original magnification: 200×). (F) Immunofluorescence staining for CD31 in tumors of the CR-PDT and control groups (original magnification: 400×). (G) Bio-TEM analysis of the morphology and structural characteristics of tumor vessels of the CR-PDT and control groups (arrowheads, enlarged endothelial spaces on the vascular wall). (H) [^18^F]F-MISO PET/CT evaluation of tumor hypoxia in the CR-PDT and control groups. (I) Semi-quantitative analysis of TBRliver of SUVmax based on [^18^F]F-MISO PET/CT (*n* = 3, mean ± SD, *p < 0.05, unpaired two-tailed t test). (J) Immunofluorescence staining for HIF-1α in tumors of the CR-PDT and control groups (original magnification: 600×).

Based on the results observed in Figure 5G, a series of examinations were performed to determine whether ECM was modulated as a result of CR-PDT. First, [^99m^Tc]Tc-FAPI was synthesized to evaluate FAP expression in tumors by using single-photon emission computed tomography (SPECT)/CT (Figure S24). At day 15 post-injection, the SUVmax value of tumors in the CR-PDT group was reduced by 50%, compared with that in the control group, indicating a significant downregulation of FAP expression in the CR-PDT group (Figures 6A and S25). This finding was further confirmed through immunofluorescence staining of FAP and α-smooth muscle actin (α-SMA), both of which are biomarkers of activated CAFs (Figures 6B and S26). These results indicate that CAFs in tumors receiving CR-PDT were deactivated or even destroyed.

Actually, dysfunctional CAFs not only impede the formation, secretion, and cross-linking of fibrous collagen, but they also exert a far-reaching impact on the morphological or rheological properties of the ECM, which is a major contributor to tumor stiffness.^36^ Therefore, we evaluated tumor stiffness in different groups using ultrasonic elastography. The results showed that tumor stiffness increased during tumor growth in the absence of intervention (Figure S27). In contrast, the tissue stiffness of tumors treated with CR-PDT (38.50 kPa) was considerably decreased, compared with that of the control group (64.90 kPa) (Figure S28). Moreover, the vessels in tumors of the CR-PDT group were larger and more filled, while flimsy and tiny vessels were found in the control group (Figure 6C). As mentioned above, tumor stiffness is mainly determined by collagen deposition and cross-linking, which constrain the fluidity of ECM components, such as hyaluronic acid (HA) and versican.^37^ Therefore, Masson’s trichrome stain, Picrosirius red (PSR) stain, and immunofluorescence double staining for HA-binding protein 1 (HABP1) and chondroitin sulfate proteoglycan 2 (CSPG2) were performed. The content of collagenous fiber, HA, and versican in tumors treated with CR-PDT was remarkably reduced, compared with that in the control group, indicating a suppressed desmoplastic response in the tumor ECM (Figures 6D, 6E, and S29). Thus, the tumor stroma was substantially degraded by the targeted interference of CR-PDT on CAFs.

Next, immunofluorescence staining for CD31 was conducted on tumors to evaluate the change of tumor vessels after the attenuation of the dense stromal barrier (Figure 6F). Consistent with the ultrasonic elastography imaging (Figure 6C), the morphology and structure of intratumoral vessels in the CR-PDT group tended to be more expanded, and more abundant dilated lumens were found in the CR-PDT group compared with the control group. Next, the fine structure of vessels was observed under bio-TEM. In CR-PDT-treated tumors, numerous porous and leaky vascular endothelial spaces were detected on the vascular wall, through which the TPZ molecules can presumably penetrate and diffuse in the region of tumor cells. In contrast, only minor voids were detected in the walls of collapsed vessels in tumors of the control group, which would severely limit the penetration of TPZ molecules (Figure 6G). Tumor stiffness is a multifactorial trait dynamically regulated by cellularity, ECM deposition, and interstitial fluid pressure.^38^ The reduction in PANC-1 cells and CAFs, along with collagen and hyaluronan degradation, may collectively contribute to tumor stiffness and vascular alterations.

As speculated above, another mechanism indicating that CR-PDT enhances the antitumor effect of TPZ may be the intensified tumor hypoxia caused by CR-PDT. To evaluate the hypoxic microenvironment of tumors treated with CR-PDT without TPZ administration, [^18^F]F-misonidazole ([^18^F]F-MISO)-based PET/CT imaging was performed after three courses of CR-PDT. The synthesized [^18^F]F-MISO exhibited a high labeling rate (Figure S30) and displayed a highly selective concentration in the tumor site with relatively stable SUVmax and SUVmean values within 3 h post-injection (Figures S31 and S32). The TBRliver of SUVmax in the tumors of the CR-PDT group was 2.20 times that of the control group, indicating a larger proportion of hypoxic tumor cells in the CR-PDT group (Figures 6H and 6I). Hypoxic conditions stimulate the release of HIF-1α.^39^ Therefore, immunofluorescence staining for HIF-1α was performed. The expression level of HIF-1α was much higher in tumors of the CR-PDT group compared with the control group (Figures 6J and S33). Thus, CR-PDT resulted in a severely hypoxic TME, with its oxygen consumption efficiently surpassing any potential increase in oxygen import from dilated vessels. These results supported the speculation that FAPI-targeted CR-PDT degrades the tumor stroma by inhibiting CAFs from secreting collagen fibrils, disrupting fibril deposition and cross-linking and limiting the formation of HA and versican. This reduces tumor stiffness and reopens collapsed vessels, allowing more TPZ molecules to perfuse the tumor bed. Moreover, the exacerbated hypoxia in the TME caused by oxygen consumption of CR-PDT further improves TPZ efficiency. The combination of CR-PDT and TPZ thus ultimately achieves optimal cancer treatment outcomes.

To confirm the effect of CR-PDT on intensifying treatment efficacy, tumors treated with TPZ, [^68^Ga]Ga&TPZ (without TiO_2_ NPs), or CR-PDT&TPZ (with TiO_2_ NPs) were examined. As shown in Figure S34, tumors treated with TPZ and [^68^Ga]Ga&TPZ showed similar high expression of FAP and α-SMA and abundant infiltration of collagenous fiber, compared with the control group, while tumors treated with CR-PDT&TPZ exhibited similar down-expressed FAP and α-SMA and reduced collagenous fibers as those in the CR-PDT group. The tumor vasculature in the TPZ and [^68^Ga]Ga&TPZ groups was slim, while that in the CR-PDT&TPZ group was mechanically decompressed and expanded as observed in the CR-PDT group. In the CR-PDT&TPZ group, HIF-1α was expressed at high levels, compared with that in the control, TPZ, and [^68^Ga]Ga&TPZ groups. Together, these findings further support our proposed mechanism of FAPI-targeted CR-PDT with TPZ for tumor treatment.

### Exploration of the mechanism of tumor ECM modulation by CR-PDT, using RNA-seq

**Figure 7.**
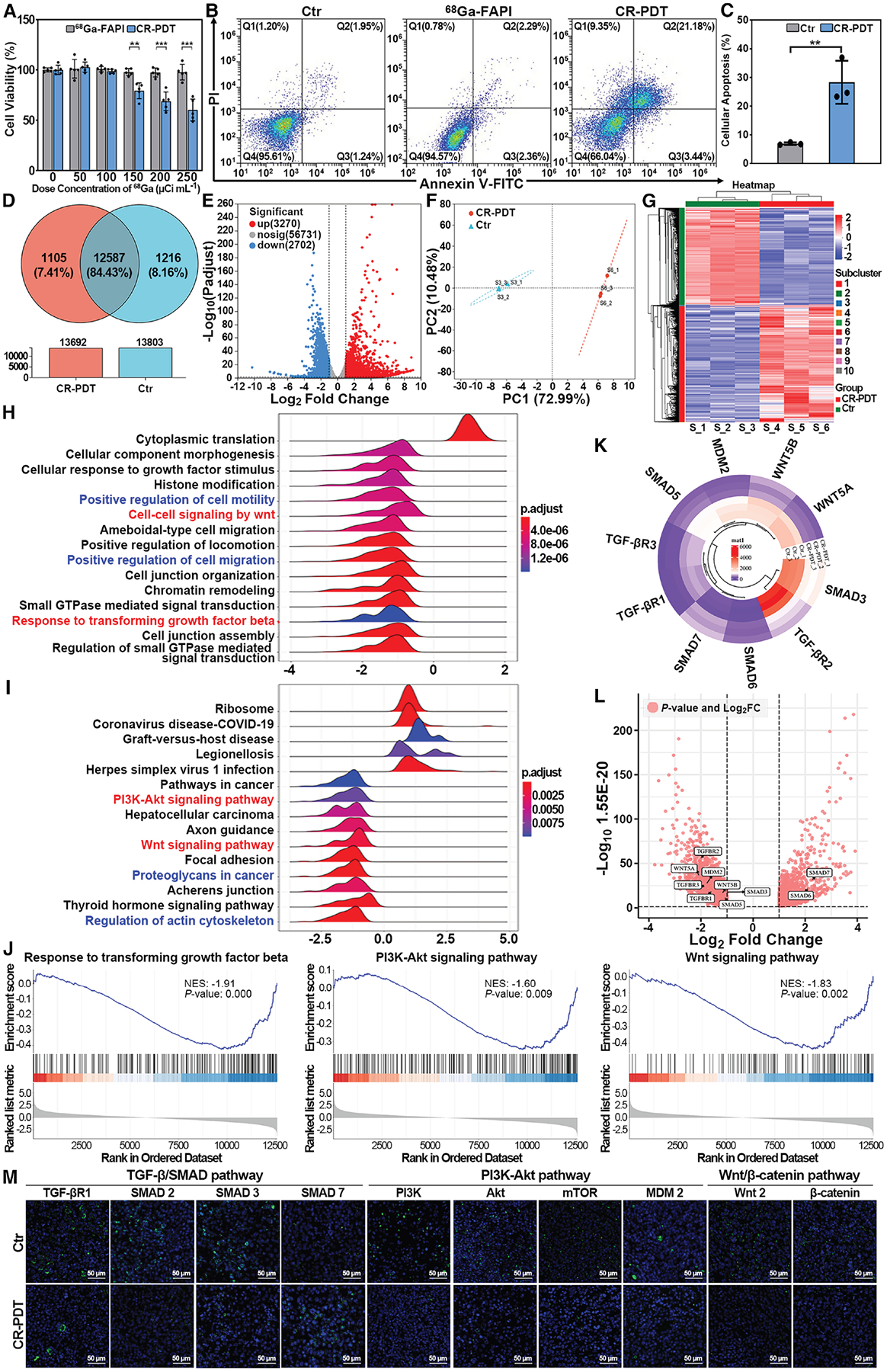
*In vitro* therapeutic evaluation and RNA-seq analysis in CR-PDT-treated CAFs and tumor immunofluorescence analysis (A) CCK-8 assay testing the killing efficiency of CR-PDT on CAFs (*n* = 5, mean ± SD, **p < 0.01, ***p < 0.001, unpaired two-tailed t test). (B) Flow cytometry analysis of cell apoptosis of CAFs treated with DMEM, [^68^Ga]Ga, or CR-PDT. (C) Quantitative comparison of apoptosis rates in different groups (*n* = 3, mean ± SD, **p < 0.01, Mann-Whitney U test). (D) Venn diagram of DEGs in CAFs treated with CR-PDT and untreated CAFs. (E) Volcano plot displaying DEGs between cells in the CR-PDT and control groups. (F) PCA of DEGs in the CR-PDT and control groups. (G) Heatmap overview of DEGs induced through CR-PDT or DMEM (control) based on cluster analysis. (H) Ridge plots of GO-based GSEA between CAFs treated with CR-PDT and those without. (I) Ridge plots of KEGG-based GSEA between CAFs treated with CR-PDT and those without. (J) GSEA of TGF-β, PI3K-Akt, and Wnt signaling pathways in CAFs. (K) Circular heatmap displaying DEGs related to the TGF-β, PI3K-Akt, and Wnt signaling pathways between CAFs in CR-PDT and control groups. (L) Volcano plot highlighting DEGs related to the TGF-β, PI3K-Akt, and Wnt signaling pathways. (M) Immunofluorescence staining for representative regulators related to the TGF-β/SMAD, PI3K-Akt, and Wnt/β-catenin signaling pathways in tumor slices collected on day 15 after different treatments (original magnification: 200×).

We further explored the underlying mechanism of how CR-PDT influences CAFs, by using *in vitro* analyses. Cell Counting Kit-8 (CCK-8) assay showed that [^68^Ga]Ga alone caused negligible damage to CAFs, but a dose-dependent killing effect was observed when [^68^Ga]Ga was combined with TiO_2_ NPs (Figure 7A). CR-PDT triggered by 7.4 MBq (200 μCi) of [^68^Ga] Ga caused 31.27% of CAFs’ death. Flow cytometry with annexin V-FITC/PI staining indicated that cell apoptosis in the CR-PDT group was 4.10 times that of the control group (Figures 7B and 7C). Integrating these results with those in Figure 6B suggests that CR-PDT deactivates or kills CAFs. The direct killing effect of CR-PDT on CAFs and the combined effect with TPZ were verified in CCK-8 assay (Figure S35). Similar to the result in PANC-1 cells in Figure 4C, CR-PDT&TPZ had the greatest deleterious effects on CAFs’ viability (36.65%), compared with CR-PDT (69.64%) or TPZ (71.09%). These results suggest that the ROS generated by CR-PDT directly kill CAFs, and the killing efficacy of CR-PDT in combination with TPZ was greatly enhanced, compared with single treatments.

To further explore the potential antitumor mechanisms of CR-PDT, RNA sequencing (RNA-seq) of CAFs treated with CR-PDT and untreated CAFs was performed, and differentially expressed genes (DEGs) were identified. Venn diagram analysis revealed that 15.57% of genes were altered in CAFs after treatment with CR-PDT (Figure 7D). Volcano plots indicated that 5,972 genes exhibited significant differences in expression in the CR-PDT group relative to the control group, with 3,270 upregulated and 2,702 downregulated genes (Figure 7E). Principal-component analysis (PCA) clearly separated the samples into two clusters, validating a distinct directionality between the two groups on the basis of similarities in gene expression (Figure 7F). The heatmap based on DEG cluster analysis displayed significant differences in genes in CAFs treated with CR-PDT, compared with controls (Figure 7G).

Gene set enrichment analysis (GSEA) with Kyoto Encyclopedia of Genes and Genomes (KEGG) and Gene Ontology (GO) analyses were carried out to further identify signaling pathways of the DEGs in CAFs treated with CR-PDT. Multiple pathways were found to be altered by CR-PDT in CAFs, among which we paid particular attention to the TGF-β, phosphoinositide 3-kinase/Akt (PI3K/Akt), and Wnt pathways (Figures 7H and 7I). Additionally, we also focused on enriched biological processes including the motility and migration of CAFs, regulation of the actin cytoskeleton, and proteoglycan generation. GSEA showed that both the highlighted pathways (Figure 7J) and biological processes (Figure S36) were negatively regulated (all with normalized enrichment score [NES] < 0, adjusted *p* < 0.05) in the CR-PDT group. These findings supported our hypothesis that CR-PDT suppresses CAFs and modulates the tumor ECM component. Further, representative regulators (TGF-βRI, TGF-βRII, TGF-βRIII, Smad3, Smad5, Smad6, Smad7, PI3K, Mouse Double Minute 2 [MDM2], Wnt5A, and Wnt5B) of the aforementioned pathways were identified and displayed in a circular heatmap; most DEGs, except for Smad6 and Smad7 genes, were downregulated in CR-PDT-treated CAFs (Figure 7K), with significant differences in DEGs as shown in the volcano plots (Figure 7L).

Finally, several representative proteins in the pathways identified above were then examined in tumors of mice from various treatment groups, using immunofluorescence analysis. The expression levels of TGF-βRI, Smad2, Smad3, PI3K, Akt, mechanistic target of rapamycin (mTOR), MDM2, Wnt2, and β--catenin were downregulated in tumors with CR-PDT treatment, and Smad7 was upregulated (Figures 7M and S37). This is consistent with the alterations of signaling pathways. To further validate the RNA-seq results, we analyzed gene expressions of the selected factors in CAFs via polymerase chain reactions (PCRs). As shown in Figure S38, the changes in mRNA expression of these proteins in CAFs treated with CR-PDT were consistent with the immunofluorescence staining and RNA-seq findings.

TGF-β is a regulatory factor of ECM biosynthesis and collagen fiber deposition.^40^ Downregulation of the downstream Smad proteins (e.g., Smad2/3, Smad4) in the TGF-β pathway deactivates CAFs and reduces the dense stroma in pancreatic tumors.^41^ Smad7 plays an antifibrotic role by inhibiting the TGF-β pathway.^42^ Suppressing the PI3K/Akt/mTOR signaling pathway is an efficient approach for ameliorating pancreatic fibrosis,^43^ with MDM2 and p53 considered important regulators of the Akt pathway.^44–46^ The Wnt/β-catenin signaling pathway is vital for mediating CAF activation and contributing to antifibrotic effects,^47^ where the downregulation of Wnt2 is associated with reduced survival of pancreatic tumors.^48^ Many previous studies have revealed the significant roles of TGF-β, PI3K/Akt, and Wnt/β-catenin signaling pathways in collagen deposition, ECM formation, and interstitial fibrosis in pancreatic tumors.^49^ The regulatory factors of these pathways are also closely linked to tumor invasion and metastasis by interfering with epithelial-mesenchymal transition (EMT) during tumor progression.^50^ These pathways have complex interactions in the fibrosis-related process, with the TGF-β pathway playing a predominant and crucial role. Both the PI3K/Akt and Wnt/β-catenin pathways crosstalk with TGF-β signaling, collectively promoting the progression of interstitial fibrosis.^49^ Together, these findings suggest that CR-PDT modulates multiple signaling pathways to influence tumor progression and treatment response; among these pathways, the downregulation of TGF-β/SMAD, PI3K-Akt, and Wnt/β-catenin pathways in CAFs may be associated with the reduced tumor fibrosis by CR-PDT and subsequently improved chemotherapy efficacy of TPZ.

### Conclusions

**Figure 8.**
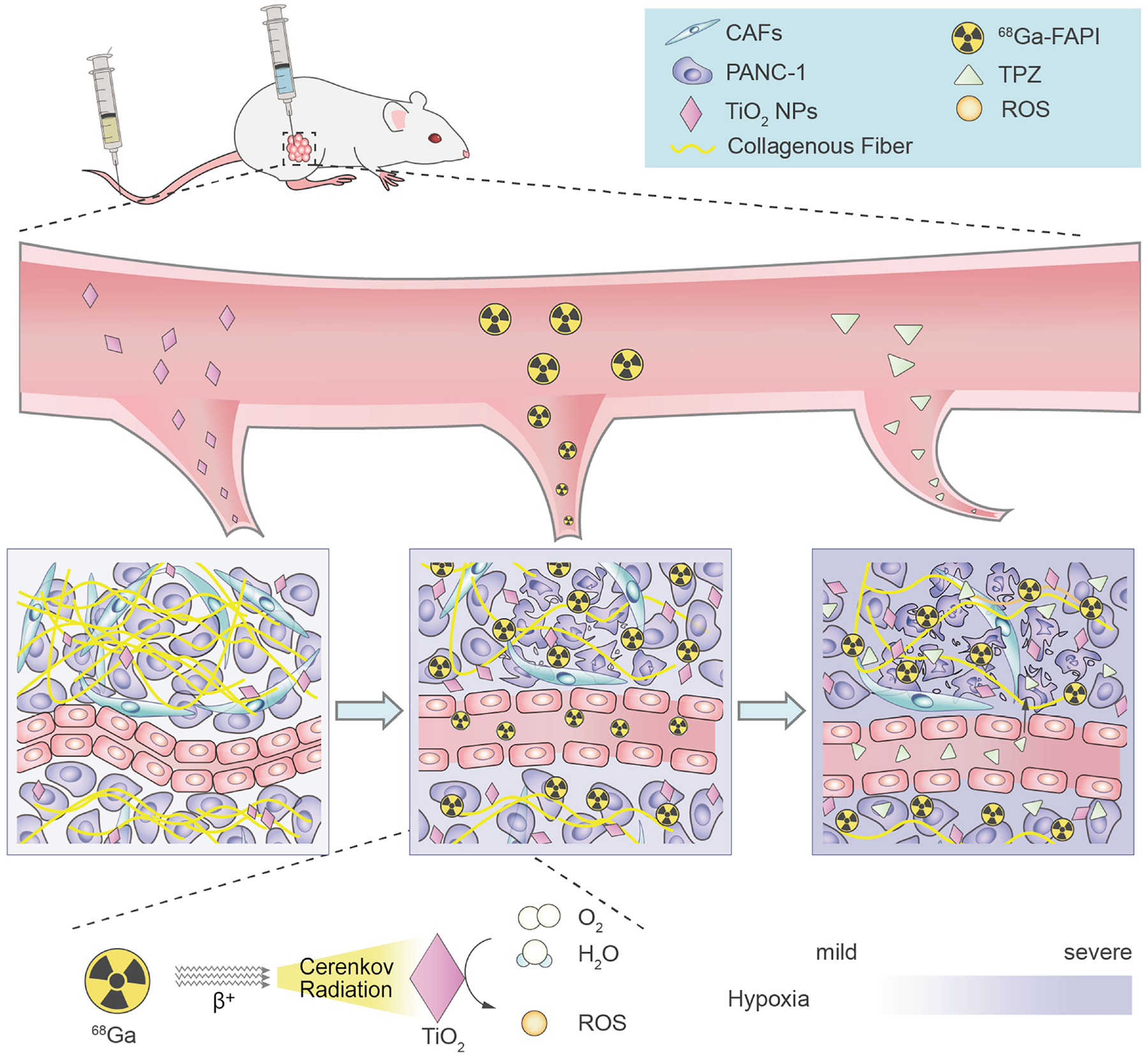
Schematic illustration of CR modulating the ECM, using [^68^Ga]Ga-FAPI and TiO_2_ NPs in combination with TPZ for improved pancreatic cancer therapy The treatment consists of three steps: (1) intratumoral injection of TiO_2_ NPs, (2) intravenous injection of ^68^Ga-FAPI, and (3) intravenous injection of TPZ.

Pancreatic cancers are characterized by robust desmoplastic stroma with excessive fibrous collagen in the ECM, which impedes drug diffusion into the tumor bed and leads to poor therapeutic outcomes. Here, we used CR emitted from [^68^Ga]Ga as an internal excitation source for TiO_2_ NPs to induce CAF-targeted CR-PDT (Figure 8). On the one hand, ROS generated through CR-PDT directly damages the tumor cells. On the other hand, CR-PDT was found to suppress the TGF-β/SMAD2/3, PI3K/Akt, and Wnt/β-catenin signaling pathways, which may be involved in inhibiting the activation of CAFs. This may be closely associated with the reduced formation and cross-linking of collagen fibrosis and degradation of the ECM. By relieving the compression caused by the stiff ECM, the previously collapsed vessels may become dilated and leaky, allowing sufficient TPZ molecules to enter and permeate the tumor bed. Meanwhile, CR-PDT exacerbated tumor hypoxia through oxygen consumption, thus enhancing the efficacy of the hypoxia-activated and bio-reductive TPZ. Both *in vitro* and *in vivo* data confirmed the improved therapeutic effect of combining CR-PDT with TPZ, compared with either treatment alone. Our proposed strategy provides new perspectives on the application of radiotracers and encourages further exploration in cancer therapy, particularly for cancers with desmoplastic stroma and excessive fibrosis.

## METHODS

Further details regarding the methods can be found in the supplemental information.

### RESOURCE AVAILABILITY

#### Lead contact

Requests for further information and resources should be directed to and will be fulfilled by the lead contact, Dalong Ni (ndl12353@rjh.com.cn).

#### Materials availability

All unique/stable reagents generated in this study are available from the lead contact with a completed materials transfer agreement.

#### Data and code availability

All data generated or analyzed during this study are included in this published article and its supplemental information. The source data that support the findings of this study are available from the corresponding author upon reasonable request.

## Supplementary Material

1

SUPPLEMENTAL INFORMATION

Supplemental information can be found online at https://doi.org/10.1016/j.celbio.2025.100221.

## Highlights

- CR of clinic ^68^Ga activates TiO_2_ nanosensitizers to damage pancreatic cancer CAFs
- Decreased CAFs modulate ECM to improve tirapazamine delivery to pancreatic cancer
- Oxygen consumption of CR-PDT enhances therapeutic effect of tirapazamine

## THE BIGGER PICTURE

Pancreatic tumors are distinguished by excessive extracellular matrix (ECM) with robust fibrosis, severe hypoxia and collapsed vessels, which are responsible for chemotherapy resistance. Cancer associated fibroblasts (CAFs) dominate the formation and cross-linking of fibrous collagen. In this study, we present a CAFs-targeted tumor treatment strategy based on Cerenkov radiation-mediated photodynamic therapy (CR-PDT). The combination of clinic [^68^Ga]Ga-FAPI and TiO_2_ nanosensitizers makes damage to CAFs, and effectively impedes collagen production, disrupts tumor stroma and decompresses vessels, thereby improving the blood perfusion and delivery of tirapazamine. In addition, the therapeutic effect of tirapazamine, a hypoxia-activated drug, is further enhanced by the oxygen consumption resulting from CR-PDT. This work presents a nanomaterial and clinical radionuclide-based strategy for enhanced treatment of desmoplastic solid tumors, particularly those with extensive fibrous stroma.
