# Supplementary material for: Cerenkov radiation modulates the extracellular matrix for improved pancreatic cancer chemotherapy
Source: Cell Biomater. Author manuscript; Available in PMC 2026 Jul 9. (PMC13345483; doi:10.1016/j.celbio.2025.100221)
Supplement: 1 [file NIHMS2126752-supplement-1.pdf]

CELBIO, Volume *2*

Supplemental information

Cerenkov radiation modulates the extracellular matrix for improved pancreatic cancer chemotherapy

Weiwei Su, Han Wang, Shuai Zhao, Tao Wang, Jessica C. Hsu, Xiuru Ji, Shuping Li, Changjing Zuo, Weibo Cai, and Dalong Ni

Supplemental Methods

Chemical agents: Tetrabutyl titanate ($C_{16}H_{36}O_4Ti$) was purchased from Sigma-Aldrich Chemical Co., Ltd. Ethyl alcohol (C_2H_6O), methyl alcohol (CH_3OH), ammonium hydroxide ($NH_3{\cdot}H_2O$), polyetherimide (PEI), sodium acetate (NaOAc) and hydrochloric acid (HCl) were obtained from Sinopharm Chemical Reagent Co., Ltd. [^{68}Ga]Ga was eluted from [^{68}Ge]Ge/[^{68}Ga]Ga generator in Changhai hospital. DOTA-FAPI-04 was provided by Zhejiang Tan Zhen Biotechnology Co., Ltd (www.zjprobe.com). [^{18}F]F-FDG was purchased from Shanghai Xinke Pharmaceutical Co., Ltd. [^{18}F]F-MISO was provided by Shanghai Ruijin hospital. Tirapazamine (TPZ) was purchased from Shanghai macklin biochemical Co., Ltd.. All above agents were used without further purification.

Bioreagents: PANC-1 cells and dulbecco's modified eagle medium (DMEM) were purchased from Shanghai Ze Ye Biotech Co., Ltd. Human pancreatic cancer-associated fibroblasts (CAFs) and relevant complete culture medium were purchased from Shanghai Kang Lang Biotech Co., Ltd. Fetal bovine serum (FBS), trypsin, phosphate-buffered saline (PBS), penicillin-streptomycin solution and Total RNA Extraction Reagent TRIeasy™ were obtained from Shanghai Yeasen Biotech Co., Ltd. Cell counting kit-8 (CCK-8) kit, Annexin V-FITC/PI apoptosis detection kit, reactive oxygen species (ROS) assay kit and γ-H_2AX kit were bought from Shanghai Yeasen Biotech Co., Ltd. Moreover, 4% Paraformaldehyde and all antibodies for immunofluorescence assays and pathological examinations were offered by Wuhan servise biotechnology Co., Ltd. The BBoxiProbe® O91 kit was provided by Shanghai Beibo biotechnology Co., Ltd.

Bioinformatic analysis. All data for FAP expression analysis in PAAD patients were based on The Cancer Genome Atlas (TCGA) and Human Protein Atlas (HPA) database. In detail, the analysis of FAP mRNA level in various tumors and corresponding normal tissue was based on Tumor IMmune Estimation Resource (TIMER) database, and the analysis of FAP protein in various tumors and adjacent normal tissue was based on University of ALabama at Birmingham CANcer data analysis Portal (UALCAN, http://ualcan.path.uab.edu/index.html). The immunohistochemical images of FAP in PAAD and normal pancreatic tissue were obtained from HPA (https://www.proteinatlas.org), and the staining intensity was grouped as not detected, low expression, medium expression and high expression, corresponding to different levels of FAP protein. The comparison of mRNA expression level of FAP in PAAD and normal pancreatic tissue in box diagram was using Mann–Whitney *U* test, with the threshold of $P < 0.01$ and $|\log_2 FC| > 1$. Prognostic value of FAP for the overall survival of PAAD patients was based on Kaplan-Meier analysis, and *p* value was given by log-rank test. Comparison for the FAP expression between tumors at T1-2 and T3-4 stage was based on *t*-test, and comparison for the FAP level among tumors of grade I, II and III-IV was based on one-way analysis of variance (ANOVA). Protein-protein interaction (PPI) network of FAP with tumor fibrosis-related proteins was constructed through the Search Tool for the Retrieval of Interacting Genes (STRING) database (http://string-db.org/). Scatter plots for the correlation between FAP and the concerned proteins were analyzed based on UALCAN database *via* the Spearman's rank correlation test.

Synthesis of TiO_2 NPs and TiO_2-NH_2. First, Tetrabutyl titanate (1 mL), ethyl alcohol (10 mL), and methyl alcohol (10 mL) were mixed homogeneously for 10 min under the room

temperature to obtain “mixture A”. Next, ethyl alcohol (50 mL), methyl alcohol (50 mL) and ammonium hydroxide (1 mL) were mixed homogeneously for 10 min under the room temperature to obtain “mixture B”. Then, the “mixture A” was added into “mixture B” drop by drop, and magnetically stirred under the room temperature for 2 hours. The final mixture was then transferred to a high pressure reactor, and maintained at 130 °C for 110 minutes for sufficient reaction. Finally, after the action was terminated, the obtained mixture was centrifugated (8000 rpm, 10 min) and washed with ethanol for 3 times. The final product was re-suspended in ethanol and preserved at 4 °C for further use. During this process, the size of TiO_2 NPs was regulated by changing the dilution rate of ammonium hydroxide. In detail, the volume ratio of ammonium hydroxide to water was respectively controlled as 1 : 2, 1 : 1, 1 : 0.5, 1 : 0 and 1.5 : 0 to gain TiO_2 NPs with average size of 20 nm, 40 nm, 60 nm, 80 nm and 100 nm.

For amidogen modification of TiO_2 NPs (TiO_2-NH_2), the above TiO_2 NPs solution (5 mg) was dissolved in ethyl alcohol (5 mL) and received magnetic stirring at 60 °C for homogeneous dispersion. Further, polyetherimide solution (1mg mL^{-1}) was added into the reaction system and stirred for 3 hours. At last, after eliminating the excess PEI by centrifugation and washing 3 times in ethyl alcohol, TiO_2-NH_2 was obtained and stored at 4 °C for further use.

Characterization of TiO_2 NPs and TiO_2-NH_2. Morphology of AuNPs and element analysis of TiO_2-NH_2 was recognized by transmission electron microscopy (TEM, FEI talos F200x G2) operated at 200 kV, equipped with energy disperse spectroscopy (EDS, EDS super-X). The integral structure of AuNPs was observed under scanning electron microscopy (SEM, ZEISS Gemini 300). The TiO_2 NPs solution, and TiO_2 NPs incubated in

PBS or FBS for 12 h and 24 h were tested by dynamic light scattering (DLS) using a Nanotrac Wave II Q Nanoparticle Size Analyzer (Microtrac, America). X-ray diffraction (XRD) patterns were performed on a Rigaku MiniFlex 600 diffractometer at Cu Kα (λ = 1.54060 nm) with the scanning speed of 5° min^{-1} and range from 5° to 90°. Characteristic UV-Vis absorption spectra of TiO_2 NPs were obtained by an UV-Vis spectrophotometer (UV-3600, Shimadzu, Japan). Fourier transform infrared (FT-IR) spectra was tested by a Thermo Scientific Nicolet iS5 software and postprocessed by a Bruker OPUS 7.2 workstation. Zeta potentials of TiO_2 NPs and TiO_2-NH_2 were measured by Malvern Zetasizer Nano ZS90 analyzer.

[^{68}Ga]Ga-FAPI labeling rate and stability tests. Before labeling, a reactor vial of the mixture of DOTA-FAPI-04 (100 μg) with NaOAc (1 mol L^{-1}, 210 μL) was prepared. Then, [^{68}Ga]$GaCl_3$ was eluted from the [^{68}Ge]Ge/[^{68}Ga]Ga generator utilizing HCl solution (0.1 mol L^{-1}, 2 mL). For labeling, the [^{68}Ga]$GaCl_3$ eluent was transferred into the reactor vial for a labeling reaction in the water bath at 100 °C for 10 min, under the condition of *p*H = 4, during which the tubes were handly shaked every 5 min for sufficient reaction.

The radiochemical purity of [^{68}Ga]Ga-FAPI-04 (abbreviated as [^{68}Ga]Ga-FAPI) and free [^{68}Ga]$GaCl_3$ were monitored by a Flow-Count radio high performance liquid chromatography (radio-HPLC) scanner (Agilent 1260 Infinity II) with a radioactivity detector (B-FC-1000, BIOSCAN), and the mixture of water and acetonitrile (volume rate, 95: 5) as the mobile phase. Further, the *in vitro* stability of [^{68}Ga]Ga-FAPI was tested by incubating with with PBS, DMEM and 10% FBS (40 μL for each) at 37 °C for different periods of time (10 min, 30 min, 1 h, 1.5 h, 2 h).

Verification of the CRET phenomenon between [^{68}Ga]Ga and TiO_2 NPs. First, the fluorescence intensity of [^{68}Ga]$GaCl_3$, TiO_2 NPs and thire mixture was tested. We first Totally, 11 eppendorf (EP) tubes (1.5 mL) were prepared, which contained [^{68}Ga]$GaCl_3$ (2 mCi mL^{-1}, 1 mCi), TiO_2 NPs (1mg) of five sizes (20 nm, 40 nm, 60 nm, 80 nm, 100 nm), or the mixture of [^{68}Ga]$GaCl_3$ with different sizes of TiO_2 NPs for 10 minutes reaction, respectively. All tubes were detected by an In Vivo Imaging System (IVIS, VISQUE Invivo Smart-LF, Korea) to collect the optical signals with a full-band emission wavelength and blocked excitation channel.

Then, the emission spectra of TiO_2 NPs, [^{68}Ga]$GaCl_3$ and their mixture were examined. Specifically, TiO_2 NPs (1.5 mg, 200 μL) and [^{68}Ga]$GaCl_3$ (74 MBq, 2 mCi, 1 mL) was mixed for 5 min, and their emission spectra were recorded using an fluorescence spectrophotometer (model, F-7100; Hitachi) without additional excitation light.

***In vitro* experiments.** The anthropogenic pancreatic cancer (PANC-1) cells were purchased from Shanghai Ze Ye Biotech Co., Ltd., and cultured in DMEM containing 10% FBS and 1% penicillin-streptomycin solution, at the condition of 37 °C humidified atmosphere with 5% CO_2. CAFs were purchased from Shanghai Kang Lang Biotech Co., Ltd., and cultured in the complete culture medium specially produced for CAFs which was supplied by the same company. The culturing condition is also 37 °C humidified atmosphere with 5% CO_2.

***In vitro* cytotoxicity of TiO_2 NPs by CCK-8.** To prepare for a CCK-8 assay, PANC-1 cells were seeded on one 96-well plates (2×10^4 cells per well) to form a matrix with 6 columns (a concentration gradient of TiO_2 NPs) multiplying 5 lines (5 replication wells).

After the cells were adhesive for 24 h in DMEM without FBS, the TiO_2 NPs solution in DMEM was added to replace the former DMEM, with the concentration of TiO_2 NPs ranging from 37.5 μg mL^{-1} doubled to 1200 μg mL^{-1}. At 24 h post the co-incubation, CCK-8 solution was added (15 μL per well) and stained the cells for 3 h in dark, and the absorbance value was detected *via* the iMark™ microplate reader (Bio-Rad, Hercules, CA, USA) at the wavelength of 450 nm. Finally, the cell viability under each concentration of TiO_2 NPs was calculated referring to the following formula: Cell viability (%) = absorbance of experimental group/absorbance of control group × 100%.

CAFs-specific cellular uptake and competitive binding assay for [^{68}Ga]Ga-FAPI. The CAFs were plated in a 24-well plate (10^5 cells per well) for form a matrix with 5 columns multiplying 3 lines (triplicate for each column). The first column was set as a compared group by a blocking assay, and pre-treated CAFs cells for 1 h with excessive dose of FAPI, which was 100 times (10 μg) of that in another four columns. One hour later, all the five columns of CAFs were incubated with [^{68}Ga]Ga-FAPI (0.185 MBq [5 μCi] of [^{68}Ga]Ga, 0.1 μg of FAPI) at 37 °C for 3 h, 0.5 h, 1 h, 2 h and 3 h, respectively. At each time point of the incubation, the medium in corresponding group was washed and collected, and the radioactive counts by a γ-counter was recorded as $cell_{out}$. The remaining cells were lysed with NaOH (1 mol L^{-1}) for 5 min at room temperature, transferred into the EP tube after washed twice by PBS, and measured for the radioactive counts as $cell_{in}$. Finally, the uptake rate by CAFs in each group was calculated as $cell_{in}$ / ($cell_{in}$+$cell_{out}$).

CCK-8 assay testing the dose- and hypoxia- dependent characteristic of TPZ. To test the dose-dependent killing efficacy of TPZ, PANC-1 cells were planked in 96-well plate

(three replication wells, 2×10^4 cells per well) for adherent growth under normoxic conditions (O_2 partial pressure is 21%). 24 h later, the culture media was replaced with DMEM containing TPZ with varying concentrations (0, 1.25, 2.5, 3.75, 5.0 and 10.0 μg mL^{-1}, 200 μL) for additional 24 h co-culture. Similar to above method of CCK-8 assay, after the CCK-8 agent (15 μL per well) was added and stained for 3 h, the absorbance value was detected and the cell viability under each concentration of TPZ was calculated.

To compare the killing efficacy of TPZ under different oxygen content condition, the adherent PANC-1 cells was classified into control and TPZ groups (including 2.5 and 5.0 μg mL^{-1}). Cells were cultured in different incubators with normoxia condition (oxygen concentration, 21%) and hypoxic condition (oxygen concentration, 1%) for 24 h, respectively. The cells viability *via* CCK-8 assay was examined and calculated referred to above procedure.

Cellular ROS yielding and DNA damage by TPZ under normoxic and hypoxic condition. After PANC-1 cells were treated by DMEM or TPZ (2.5 μg mL^{-1}) in two six-well plates under different oxygen conditions (O_2 partial pressure of 21% or 1%) for 24 h, all cells was harvested and stained by 2',7'-Dichlorodi-hydrofluorescein diacetate (DCFH-DA) (1 mL, 10 μM, 1 : 1000 dilution in DMEM) probe for 30 min at 37 °C in dark. After removing the free probe outside cells by DMEM washing, the cells were resuspended in PBS (200 mL) and examined by flow cytometry software (ImageStream mkII, Merck-Millipore, Seattle, WA, USA).

To test the DNA DSBs caused by TPZ, PANC-1 cells grew on glass coverslips in six-well plates were treated by TPZ for 24 h under normoxic and hypoxic condition, respectively. After fixed by 4% paraformaldehyde (1.0 mL per well), cells were permeabilized with

Triton X-100 (1%, 100 μL, diluted in PBS) for 20 min, rinsed twice by PBS and blocked with 3% bovine serum albumin (BSA) for 30 min. Then the BSA was removed, and the anti-phospho-H_2AX antibody (diluted 1: 200 in PBS) was added for co-incubation overnight at 4 °C. Next, cells were washed and incubated with Alexa Fluor 488 goat anti-rabbit IgG (diluted 1: 400) for another 50 min in dark. Further, cells were rinsed and the nuclei were counterstained with DAPI for 10 min in dark. After all superfluous staining were washed and mounted, the fluorescence images were obtained by a fluorescence microscope (Nikon Eclipse CI, Japan).

Cellular uptake behavior of TiO_2 NPs in PANC-1 cells and CAFs. PANC-1 cells and CAFs were grown on coverslips in confocal dishes to adhere for 24 h. Meanwhile, FITC (2 μL, 1 mg mL^{-1}, dissolved with DMSO) was added to TiO_2 NPs (150 μg mL^{-1}) for 12 h stirring in dark. The obtained FITC-TiO_2 NPs was co-cultivated with PANC-1 cells and CAFs for 5 h. Then, the un-internalized TiO_2 NPs were removed by PBS washing, followed by fixation with 3.7% formaldehyde (in PBS) at room temperature for 15 min. Subsequently, cells were washed twice with PBS containing 0.1% Triton X-100 and then stained with DAPI (1 mL, 1:100 dilution in PBS) for 5 min in dark. After washing with PBS containing 0.1% Triton X-100, cells were stained with phalloidin-Alexa Fluor 555 (1:100 dilution in PBS with 1–5% BSA and 0.1% Triton X-100) for 45 min in the dark. Finally, cells were washed bwith PBS containing 0.1% Triton X-100 and imaged using confocal laser scanning microscopy (CLSM; Olympus FV 1000, Tokyo, Japan). FITC, DAPI and phalloidin-Alexa Fluor 555 were excited at 488 nm, 350 nm and 555 nm, respectively.

CCK-8 assay testing treatment efficacy of CR-PDT&TPZ. To comprehensively evaluate the killing efficacy of CR-PDT&TPZ, the adherent cells in 96-well plate were divided into [^{68}Ga]Ga, CR-PDT, [^{68}Ga]Ga&TPZ and CR-PDT&TPZ group, in which the dosage of [^{68}Ga]Ga varying from 1.85, 3.7, 5.55, 7.4 to 11.1 MBq mL^{-1} (50, 100, 150, 200 to 300 μCi mL^{-1}). After 24 h treatment, the cell viability was tested *via* CCK-8 assay and the histogram was drawn, and the proper treatment dosage was defined.

Cellular treatment efficiency in PANC-1 cells. For *in vitro* therapeutic experiments, PANC-1 cells were seeded onto six-well culture plates for 24 h adherent growth in DMEM. Especially, cells for γ-H_2AX staining by immunofluorescence assay were grown on the preset cell culture slides placed in wells. Later, DMEM was substituted by DMEM, or DMEM containing [^{68}Ga]Ga, TiO_2 NPs, [^{68}Ga]Ga&TiO_2 NPs, TPZ or [^{68}Ga]Ga&TiO_2 NPs&TPZ (7.4 MBq mL^{-1} [200 μCi mL^{-1}] of [^{68}Ga]Ga corresponding to 150 μg mL^{-1} of TiO_2 NPs and 2.5 μg mL^{-1} of TPZ). The treatment lasted for another 24 h.

For cell apoptosis test by flow cytometry, cells were trypsinized, centrifugated and collected from above six groups, which were resuspended in binging buffer (100 μL) and incubated with Annexin V-FITC (5 μL) and propidium iodide (PI, 5 μL) for 15 min in dark. After washed twice and diluted with PBS (400 μL), the cell apoptosis rate was analyzed using a flow cytometry analyzer (ImageStream mkII, Merck-Millipore, Seattle, WA, USA) equipped with the IDEAS™ Software (Millipore) for quantitative calculation.

The method for detecting ROS yielding by flow cytometry and DNA DSBs by immunofluorescence analysis was same as the above description in "Cellular ROS yielding and DNA damage by TPZ under normoxic and hypoxic condition".

Oxygen consumption test by CR-PDT. First, the intracellular oxygen condition was evaluated by the BBoxiProbe® O91 kit. PANC-1 cells (2×10^5 cells/well) were grown in a six-well plate to adhere for 24 h, and then treated with DMEM (control), $[^{68}Ga]Ga$ (7.4 MBq mL^{-1} [200 μCi mL^{-1}]), TiO_2 NPs (150 μg mL^{-1}) or $[^{68}Ga]Ga\&TiO_2$ NPs for another 24 h. Then, DMEM or $[^{68}Ga]Ga\&TiO_2$ NPs was removed and cells were stained with BBoxiProbe® O91 (10 μM in serum-free DMEM) for 30 min in dark. After washed twice by PBS and excited at 488 nm, the fluorescence images were obtained by a fluorescence microscope (Nikon Eclipse CI, Japan).

In a similar method with γ-H_2AX detection, cells treated with DMEM or $[^{68}Ga]Ga\&TiO_2$ NPs were first fixed by 4% paraformaldehyde (1.5 mL, 10 min at room temperature), then permeabilized with Triton X-100 (0.1% in PBS) for 20 min and blocked with 3% bovine serum albumin (BSA) for 30 min. After the BSA was removed, cells were incubated with the anti-HIF-1α antibody (diluted 1: 300 in PBS) overnight at 4 °C. Then, cells were washed and incubated with CY3-labeled goat anti-rabbit IgG (diluted 1: 300) for 50 min at room temperature. Next, the cells were rinsed by PBS (pH = 7.4) for 3 times and dried, the nuclei were counterstained with DAPI (1 μg mL^{-1}) for 10 min in dark at room temperature. After washed and dried again, the slices were mounted using antifade mounting medium. Finally, with the excitation wavelength of DAPI at 380 nm and CY3 at 560 nm, the fluorescence images were collected by a fluorescence microscope (Nikon Eclipse CI, Japan).

Cellular treatment assessment in CAFs. For the CCK-8 assay on CAFs, CAFs were planked in 96-well plate (5 replication wells, 2×10^4 cells per well) for 24 h adherent growth. Afterwards, the culture media was replaced with DMEM containing $[^{68}Ga]Ga$ or

[^{68}Ga]Ga&TiO_2 NPs (150 μg mL^{-1} of TiO_2 NPs) for additional 24 h co-culture, with varying radiation dosages of [^{68}Ga]Ga of 0, 1.85, 3.7, 5.55, 7.4 and 11.1 MBq mL^{-1} [0, 50, 100, 150, 200 and 300 μCi mL^{-1}], respectively. After 24 h incubation, CAFs was stained by CCK-8 agent (15 μL per well) for 3 h and the cell viability was detected and calculated as above method for PANC-1 cells.

To further explore the combination function of CR-PDT and TPZ on CAFs, the adherent CAFs were co-cultured with DMEM, [^{68}Ga]Ga, [^{68}Ga]Ga&TiO_2 NPs, TPZ or [^{68}Ga]Ga&TiO_2 NPs&TPZ (7.4 MBq mL^{-1} [200 μCi mL^{-1}] of [^{68}Ga]Ga corresponding to 150 μg mL^{-1} of TiO_2 NPs and 2.5 μg mL^{-1} of TPZ) for 24 h. Later, the cell viability was examined by CCK-8 assay with the similar method as above.

For cell apoptosis test by flow cytometry, the adherent CAFs were treated by DMEM, [^{68}Ga]Ga and [^{68}Ga]Ga&TiO_2 NPs for 24 h (7.4 MBq mL^{-1} [200 μCi mL^{-1}] of [^{68}Ga]Ga corresponding to 150 μg mL^{-1} of TiO_2 NPs). Then, CAFs were harvested, resuspended in binging buffer (100 μL) and stained by Annexin V-FITC (5 μL) and propidium iodide (PI, 5 μL) for 15 min in dark. After washed and resuspended in PBS (400 μL), the cell apoptosis rate was tested and calculated as above method for PANC-1 cells.

Animal model construction. All animal experiments were performed according to the guideline of Committee on Ethics of Medicine, Navy Medical University, PLA (Approval Number, **NMC2021010**). BALB/c nude mice (5 weeks, female) were provided by Shanghai Jihui Biological Technology Co. Ltd. and raised in specific pathogen-free (SPF) environment with free access to sufficient water and food. For establishment of subcutaneous pancreatic cancer model, the mixture (totally 100 μL) of cell suspension containing equal counts of PANC-1 cells (5×10^6) with CAFs (5×10^6) were

subcutaneously injected into the right posterior limb of each mouse. When the tumor volume reached approximately 100 cm^3, mice in good health condition were chosen for the following experiments.

Intratumoral retention of TiO_2 NPs by ICG-based fluorescence imaging. To obtain the ICG-labeled TiO_2 NPs (TiO_2-ICG), the prepared TiO_2-NH_2 was firstly centrifuged at 7000 r min^{-1} for 10 min to remove the liquid supernatant, and then dried in the oven at 65 °C for 1 h to get powdery TiO_2-NH_2. Further, TiO_2-NH_2 (600 μg) was resolved in the ICG-NHS solution (10 μg) and stirred overnight in the dark. After the labeling reaction finished, a centrifuge tube (50 mL) with developing solvent (20 mL, dimethyl sulfoxide: H_2O = 1: 4) was pre-balanced for 15 min. Then the immunochromatographic test strip (10 cm × 1 cm) was prepared and the tested sample (10 μL) was added on the center of 1 cm from the head of the strip, followed with infiltration for 20 min in the developing solvent. Finally, the strip was dried and imaged by the IVIS instrument (VISQUE Invivo Smart-LF, Korea).

Before the *in vivo* fluorescence imaging, TiO_2-ICG (10 μL) with varied sizes of TiO_2 NPs was subcutaneously injected into the tumor, and imaged by the IVIS instrument at different time points (7 h，24 h，48 h，72 h，120 h，168 h) post injection (P.I.) after air anesthesia. The mean fluorescence intensity was measured and calculated. At day 7 P.I., mice were executed and the tumor and important organs (heart, liver, spleen, lung, kidney) were resected and the remained fluorescence signals were acquisited by the IVIS instrument.

Intratumoral distribution of TiO_2 NPs by Cy5-based fluorescence imaging. To better visualize the intratumoral distribution of TiO_2 NPs, we administered injected Cyanine 5 amine (Cy5)-labeled TiO_2 NPs (TiO_2-Cy5, 60 nm) using a multi-point intratumoral

injection method. Tumors were dissected 1 day post-injection, and 5 μm frozen sections were prepared. Then, the paraffin-embedded sections were dewaxed, underwent heat-induced antigen retrieval (EDTA, 95°C, 20 min), and were blocked with 1% BSA. Slides were incubated with anti-CK19 antibody (1:100, 4°C overnight), and nuclei were counterstained with DAPI (1 μg/mL, 5 min). After PBS washes, slides were mounted and imaged using slide scanners (Pannoramic MIDI, Pannoramic 250 FLASH, 3DHISTECH, Hungary).

[^{68}Ga]Ga-FAPI PET/CT imaging. To evaluate the targeting ability and tumoral accumulation of [^{68}Ga]Ga-FAPI, the tumor-bearing mice was intravenously injected with [^{68}Ga]Ga-FAPI (0.37 MBq [10 μCi] per mice) *via* the tail vein, and scanned by the PET/CT at 20 min, 60 min and 90 min P.I..

***In vivo* treatment procedure and therapeutic evaluation.** To evaluating the *in vivo* treating efficiency, the tumor-bearing mice with nearly equal tumor size and good health condition were randomly divided into five groups (5 mice per group), including Control (DMEM), [^{68}Ga]Ga-FAPI, CR-PDT, TPZ and CR-PDT&TPZ group. For mice receiving CR-PDT&TPZ treatment, TiO_2 NPs (700 μg per mouse) were firstly intratumorally injected, 12 hours later, [^{68}Ga]Ga-FAPI (27.9 MBq [755 μCi] per mouse) was intravenously injected every 12 hours and repeated for 3 courses. Finally, at 12 h post the last injection of [^{68}Ga]Ga-FAPI, TPZ (50 μg per mouse) was intravenously injected to the mice tail.

During the treatment course, tumors were measured (length and width, mm) every 2 days and photoed every 4 days. The initial tumor volume (V_0) at day 0 P.I. and tumor volume (V = length × width × width × 2^{-1}) within the subsequent 15 days were recorded, and the

relative tumor volume ($V V_0^{-1}$) was calculated. Also, mice weight was recorded every 2 days, and the survival status of mice in 5 groups (8 mice per group) was recorded within the 60-day observation.

***In vivo* Imaging for therapeutic evaluation.** Herein, we adopted multiple imaging means to evaluate the treatment response of tumor. First, [^{18}F]F-FDG PET/CT was performed in mice of control and CR-PDT&TPZ group, respectively at 4 days before the treatment, at 1 day before the treatment on and at 15 days after the treatment. In detail, mice were fasted for solids and liquids overnight, and then anesthetized with 3% pentobarbital (50 - 75 μL per mouse) before intraperitoneally injected with [^{18}F]F-FDG (7.4 MBq [200 μCi] per mouse, 125 μL). About 30 minutes later, PET/CT scanning was performed and the SUVmax value was measured and recorded.

At the end of the treatment, tumors in control and CR-PDT group received [^{99m}Tc]Tc-FAPI SPECT/CT scanning. First, by utilizing $SnCl_2$ diluted by HCl as a reducing agent and ethylenediamine diacetic acid (EDDA) and Tricine as co-ligands, the Na[^{99m}Tc]TcO_4 solution (25 mCi) was incubated with HYNIC conjunct FAPI-04 (HYNIC-FAPI-04, 20 μg) in 100 °C water bath for 15-min reaction, and the [^{99m}Tc]Tc labeled HYNIC-FAPI-04, abbreviated as [^{99m}Tc]Tc-FAPI, was finally obtained. With thin layer chromatography paper as stationary phase and acetone (5 mL) as mobile phase, the labeling rate was measured. For SPECT/CT imaging, the mice were intravenously injected with [^{99m}Tc]Tc-FAPI (18.5 MBq [500 μCi] per mouse) and imaged 1 h P.I.. Furthermore, the stiffness of tumor tissue in mice of control and CR-PDT groups was detected at the end of the treatment, based on the ultrasonic elastography technique *via* an ultrasound diagnostic

imaging system (SuperSonic Imagine, Aix-en-Provence, France), and the tumor stiffness was quantified with the threshold of 30 kPa.

In addition, [^{18}F]F-MISO PET/CT imaging was also performed to evaluate the anaerobic condition of tumor before and after CR-PDT treatment. In detail, [^{18}F]F was produced and labeled with the tracer MISO to give [^{18}F]F-MISO, at 1 h after the mouse was intravenously injected with [^{18}F]F-MISO (11.1 MBq [300 μCi] per mouse), PET/CT scanning was conducted and SUVmax of TBRliver was calculated.

Pathological examinations. At 15 days P.I., the randomly selected mice from each group were sacrificed by overdose anesthetics, and tumors were dissected and sliced for pathological examination, including hematoxylin and eosin (H&E) staining, TdT mediated dUTP nick end labeling (TUNEL) and immunohistochemical analysis for Ki-67, TNF-α, BAX and C-caspase 3.

To access the ECM remodeling, the intratumoral collagenous fiber was quantified by Masson's trichrome and Picrosirius red stain, hyaluronic acid and proteoglycan was recognized by immunofluorescent staining for HA-binding protein 1 (HABP 1) and antibody to chondroitin sulfate proteoglycan 2 (CSPG2). Vessels were recognized by immunofluorescent staining for by CD31 antibody as well as bio-TEM. To indentify the activated CAFs, immunofluorescent staining for FAP and α-SMA was conducted. Moreover, the HIF-1α was analyzed by immunofluorescent staining to evaluate the hypoxic condition of tumor.

In addition, to further verify the regulated TGF-β, PI3K/Akt and Wnt pathways in CAFs, the relevant proteins in tumors treated by CR-PDT or those without treatment were

examined by immunofluorescence staining, including TGF-βRI, Smad2, Smad3, Smad7, PI3K, Akt, mTOR, MDM2, Wnt2 and β-catenin.

RNA-seq analysis of CR-PDT-treated CAFs. For RNA-seq analysis, CAFs were seeded onto six-well culture plates for 24 h adherent growth in FBS-free medium (5×10^5 cells in 1 mL medium per well, three repeated wells for both two groups). After attachment, the medium was discarded and substituted by fresh medium (1 mL) or isopyknic medium containing [^{68}Ga]Ga & TiO_2 NPs (7.4 MBq mL^{-1} [200 μCi mL^{-1}] of [^{68}Ga]Ga corresponding to 150 μg mL^{-1} of TiO_2 NPs). After 24 h treatment, CAFs from both groups were collected and total RNA was isolated by Trizol reagent. The RNA-seq was performed by Shanghai Majorbio Bio-pharm technology Co., Ltd. and the data were analyzed on the online platform of Majorbio Cloud Platform (www. majorbio. com).

Polymerase Chain Reactions (PCRs) analysis of gene expressions of selected factors in CAFs. For PCRs analysis, CAFs from the control and CR-PDT groups were collected, and total RNA was extracted using Trizol Reagent (1 mL). RNA purity was assessed using a NanoDrop 2000, and RNA integrity was verified with a Bioanalyzer (RIN >7). The RNA was then diluted to a concentration of 20 ng μL^{-1}. A 20 μL reverse transcription (RT) reaction system was prepared, consisting of: 5 × SweScript Mix (4 μL), gDNA Remover (1 μL), Total RNA (5 μL) and RNase free water (10 μL). After gentle mixing and centrifugation, the RT reaction was performed under the following conditions: 25 °C for 5 min, 42 °C for 30 min and 85 °C for 5 min. To analyze the expression of TGF-βRI, Smad2, Smad3, PI3K, Akt, mTOR, MDM2, Wnt2 and β-catenin in CAFs, specific PCR primers were re-designed. The qPCR reactions were performed in 0.1 mL PCR plates, with each 20

μL reaction system containing: 10 μL of SYBR Green qPCR Master Mix, 0.8 μL of PCR primers (10 μM each), 2.0 μL of cDNA and 7.2 μL of Nuclease-Free H_2O. After loading the samples, the plate was sealed, followed by centrifugation in a microplate centrifuge. Later, RNA was amplified with following conditions: initial denaturation (95 ˚C, 30 sec), Amplification (40 cycles) including denaturation (95°C, 15 sec) and annealing/Extension (60°C for 30 sec), and melting curve analysis based on temperature rising from 65 ˚C to 95 ˚C (increment of 0.5 °C per step, 5 sec per step). Finally, using GAPDH as the housekeeping gene, the expression levels of the target genes were quantitatively analyzed by real-time fluorescent qPCR (Bio-Rad CFX Connect).

Statistical analysis and reproducibility. The statistical analyses were performed by SPSS 21.0 software (IBM Corp., Armonk, NY, USA). All experiments were repeated independently three or five times, and the data were displayed as standard deviation (s.d.). Statistical differences between two groups were calculated by unpaired Student's *t* test for data conform to normal distribution, or a Mann-Whitney U test for non-normally distributed data. Statistical differences among three groups was assessed by one-way ANOVA followed with LSD-*t* post-hoc test for normally distributed sets with equal variance, or one-way ANOVA with Dunnett's T3 test for normally distributed sets with unequal variance, or one-way ANOVA with Kruskal-Wallis test for data does not conform to a normal distribution. The survival distributions among groups were compared using the Log-rank test (Mantel-Cox method), with pairwise comparisons adjusted by the Bonferroni correction (significance level: $\alpha = 0.01$). For other statistical analysis, the two-sided $P < 0.05$ was considered significant. In detail, P was labeled with different

number of asterisks (*) according to its actual value (*$P < 0.05$, **$P < 0.01$, ***$P <$ 0.001).

Supplemental Figures

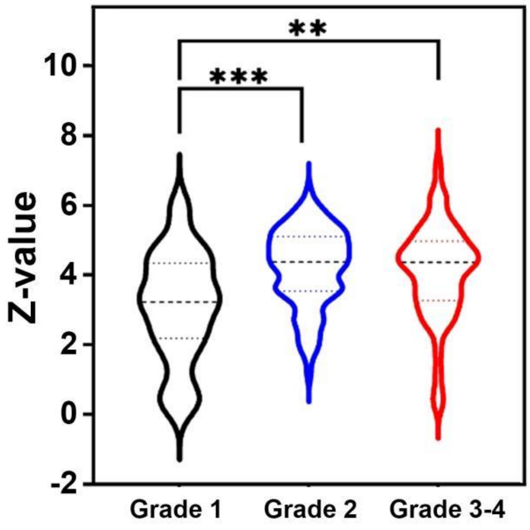

Figure S1. Expression of FAP in PAAD at different pathological grades.

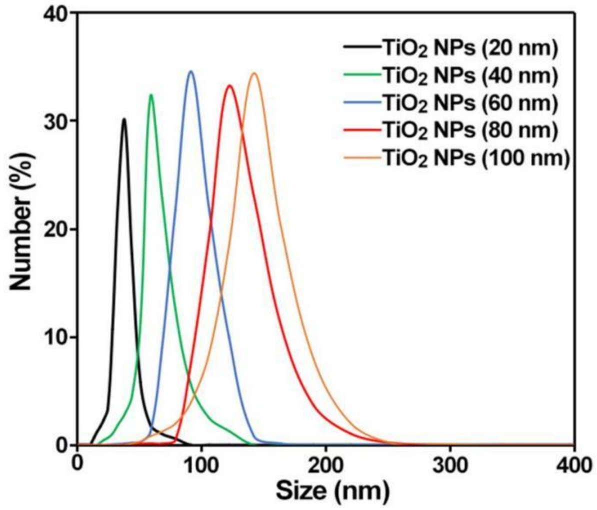

Figure S2. Hydrodynamic radius of TiO_2 NPs with different sizes.

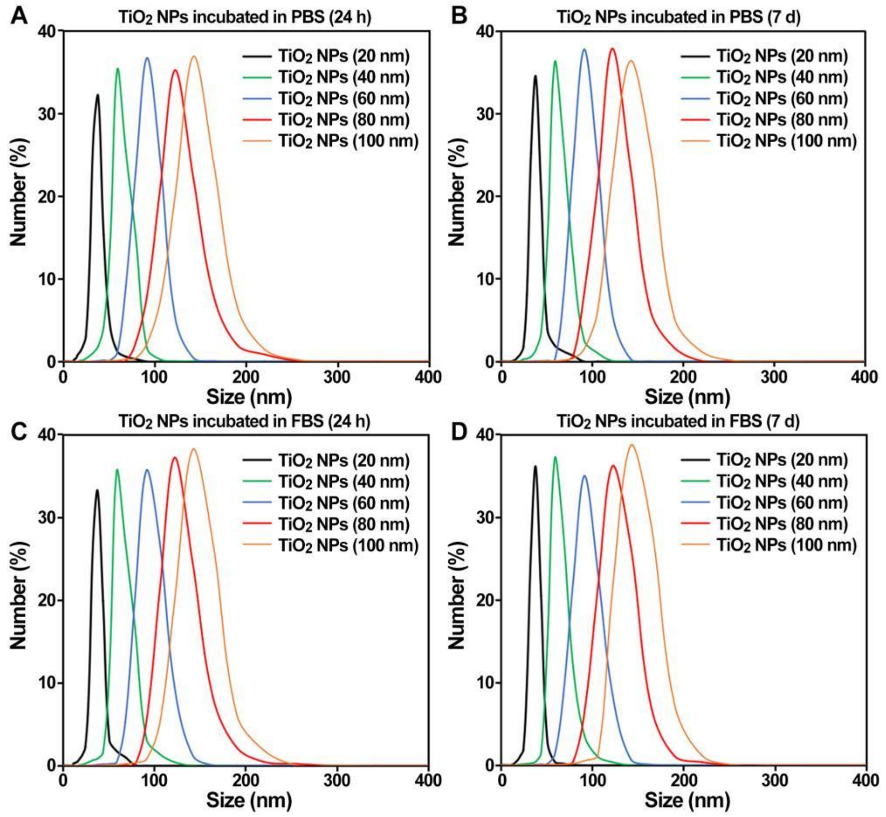

Figure S3. Hydrodynamic radius of TiO_2 NPs incubated in PBS or FBS for different time.

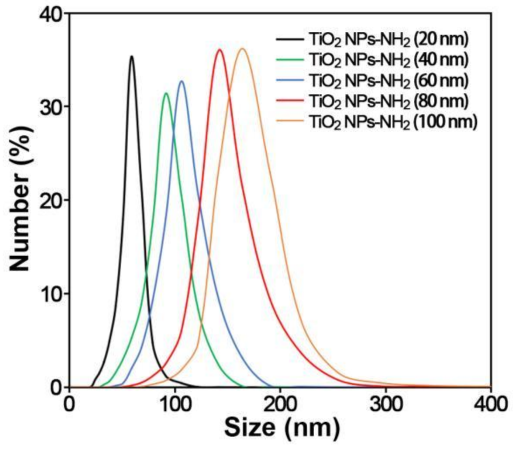

Figure S4. Hydrodynamic radius of TiO_2 NPs-NH_2 with different sizes.

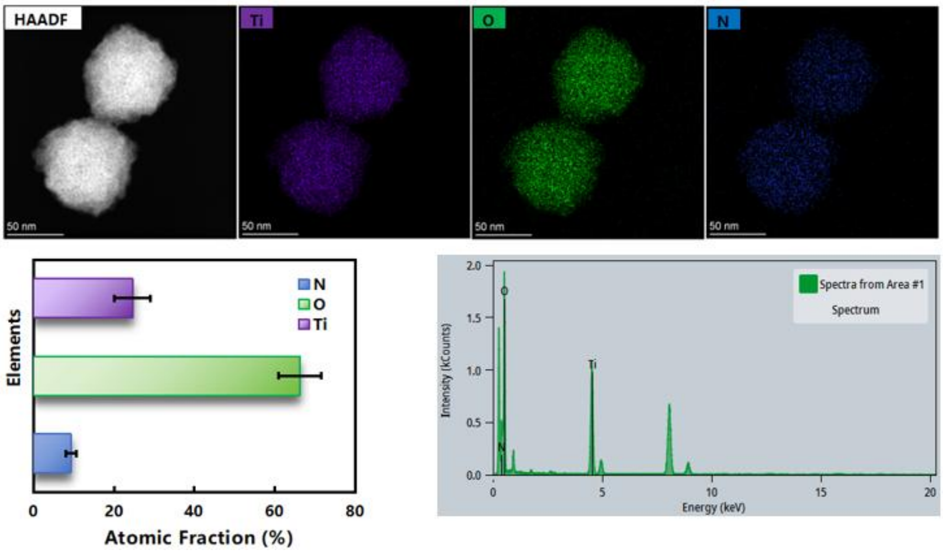

Figure S5. Element analysis for the components of TiO_2-NH_2 including Ti, O and N.

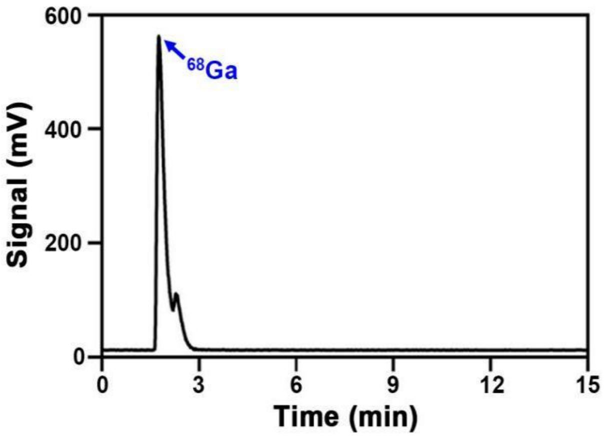

Figure S6. HPLC traces of free $^{68}Ga^{3+}$ in [^{68}Ga]$GaCl_3$.

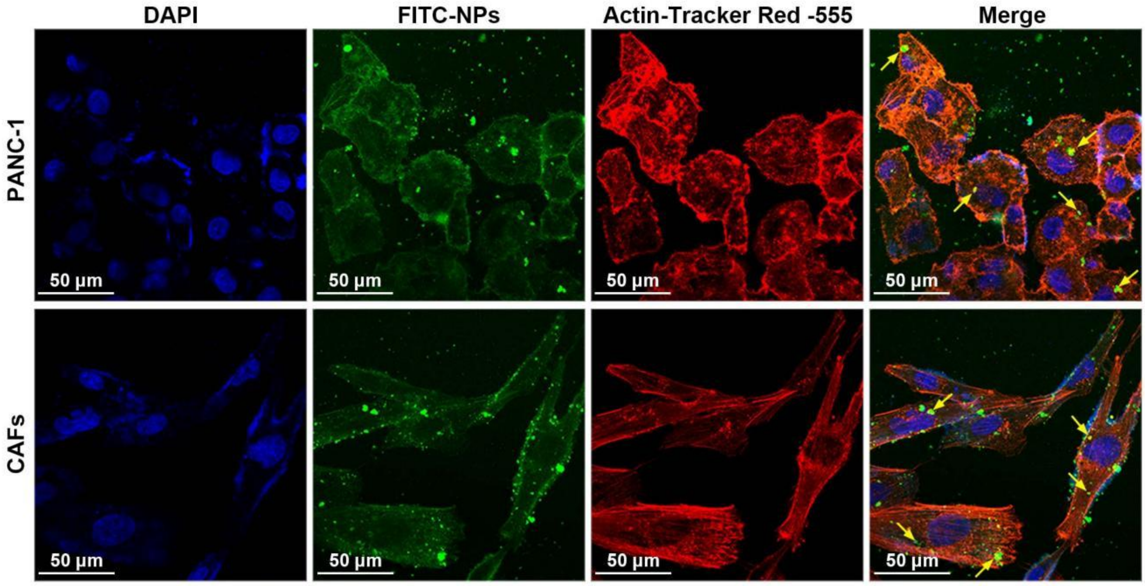

Figure S7. CLSM images showing the intracellular distribution of FITC-labeled TiO_2 NPs in PANC-1 cells and CAFs (yellow arrow, TiO_2 NPs in cytoplasm).

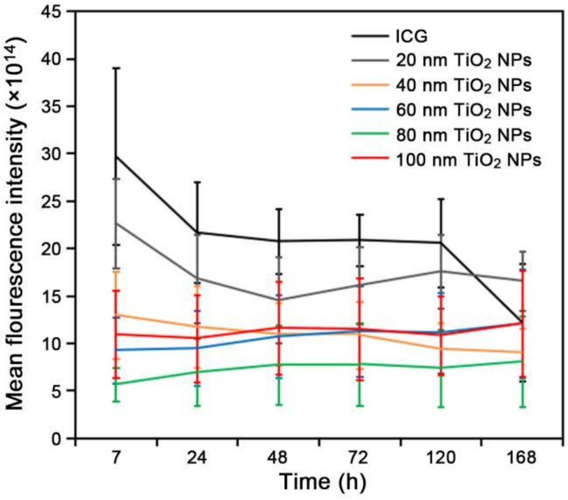

Figure S8. Quantitative analysis for mean fluorescence intensity of tumors after injected with ICG or TiO_2-ICG within 7 days.

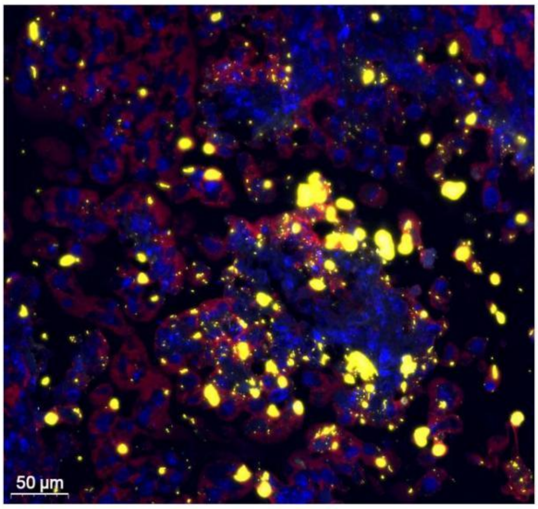

Figure S9. Distribution of TiO_2-Cy5 one day after intratumoral injection (Blue, DAPI stained cell nuclei; Red, CK19 stained cytoplasm, an epithelial-derived marker; Yellow, TiO_2-Cy5).

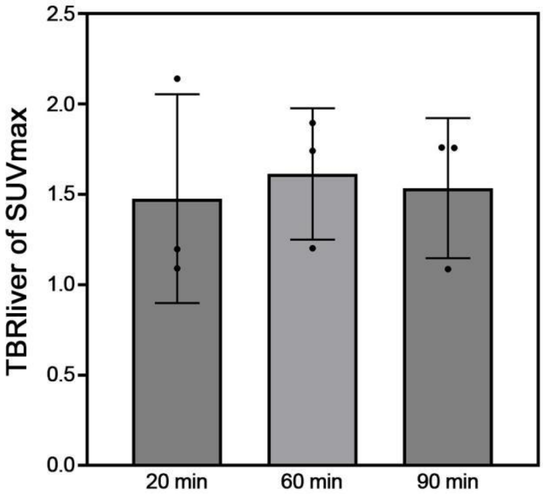

Figure S10. Semi-quantitative analysis of TBRliver based on SUVmax in ^{68}Ga-FAPI PET/CT (n = 3, mean ± s.d.).

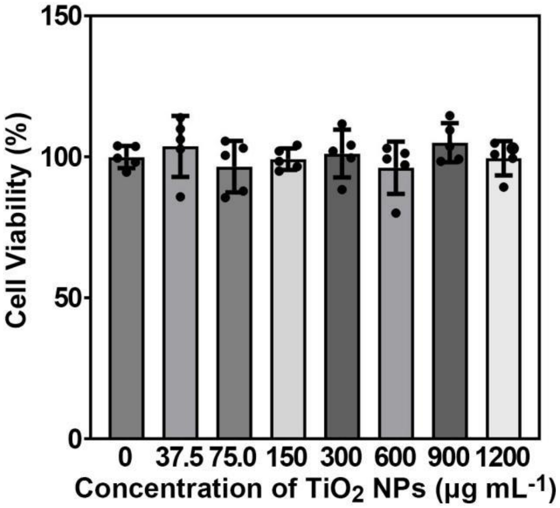

Figure S11. CCK-8 assay test of the relative viability of PANC-1 cells after 24 h co-incubation with different concentrations of TiO_2 NPs (n = 5, mean ± s.d.).

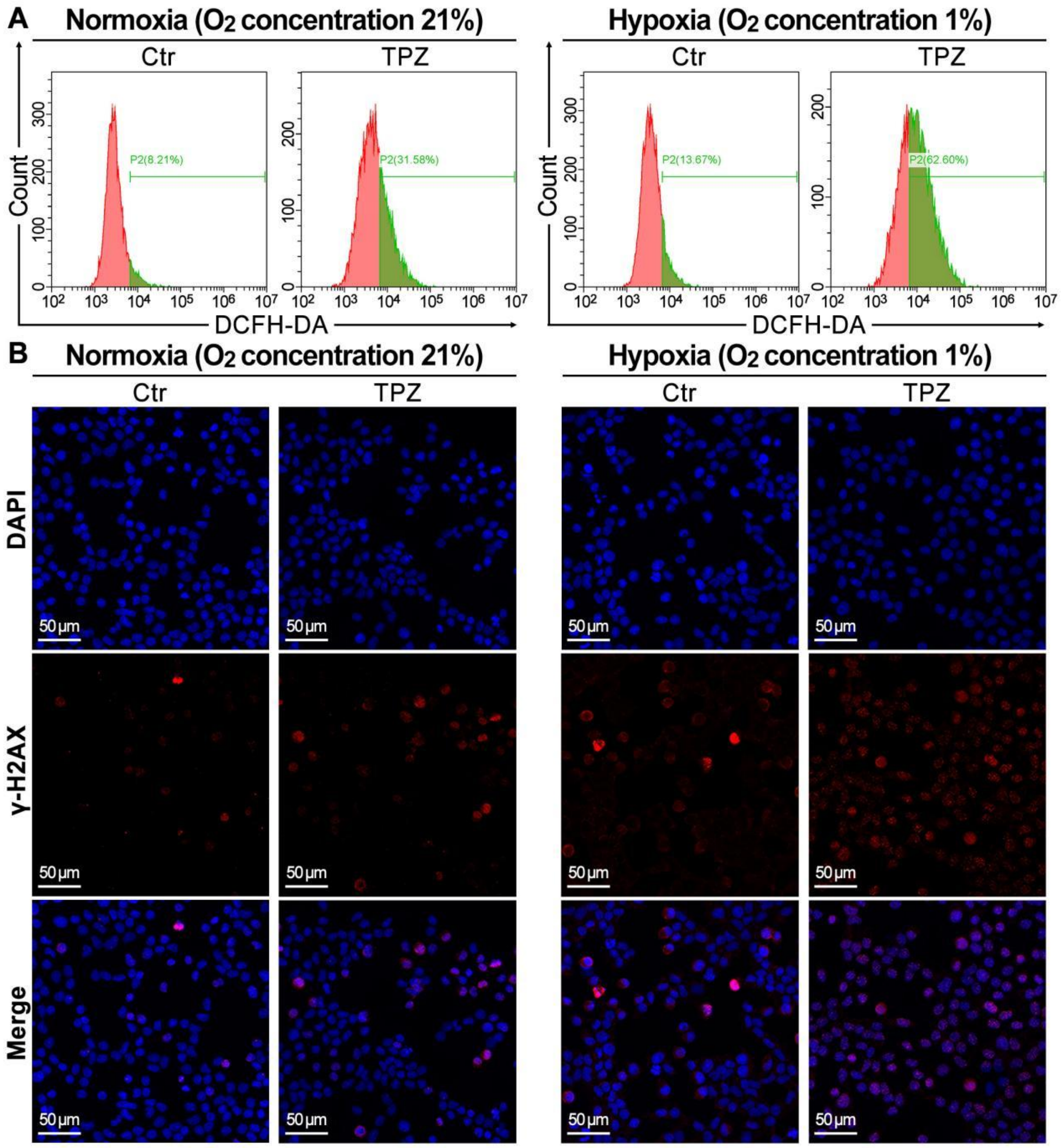

Figure S12. The cellular ROS yielding and DNA damage by TPZ under normoxic and hypoxic condition

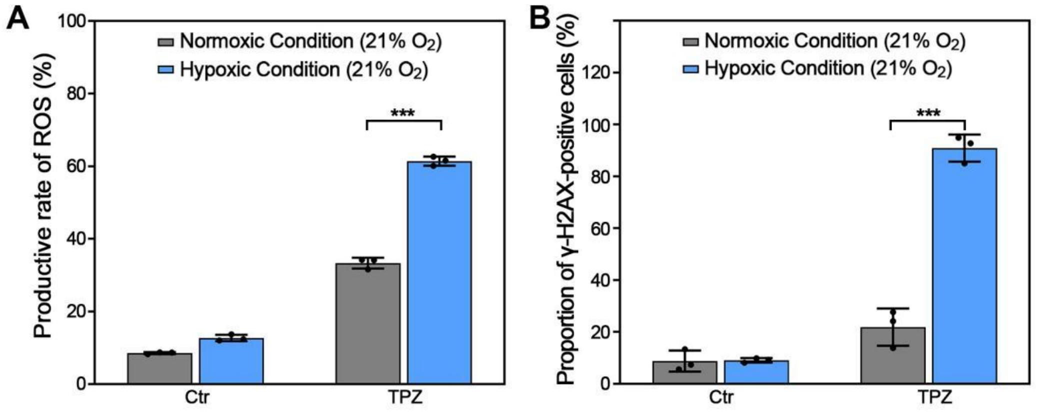

Figure S13. Quantitative analysis of ROS yielding (A) and DAN DSBs (B) by TPZ under normoxic and hypoxic condition (n = 3, mean ± s.d., ***P < 0.001, unpaired two-tailed t-test).

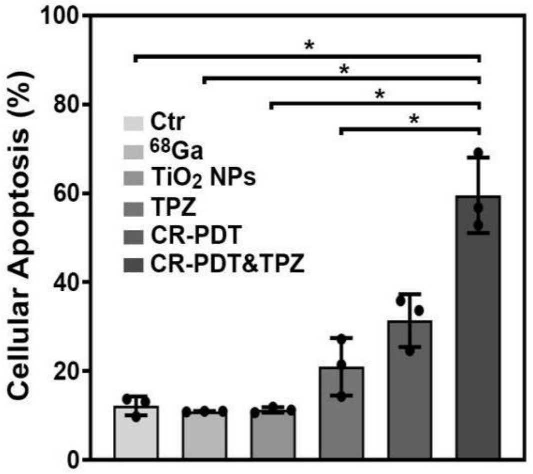

Figure S14. Quantitative comparison of the rate of cell apoptosis in different groups (n = 3, mean ± s.d., *P < 0.05, one-way ANOVA with Dunnett's T3 test).

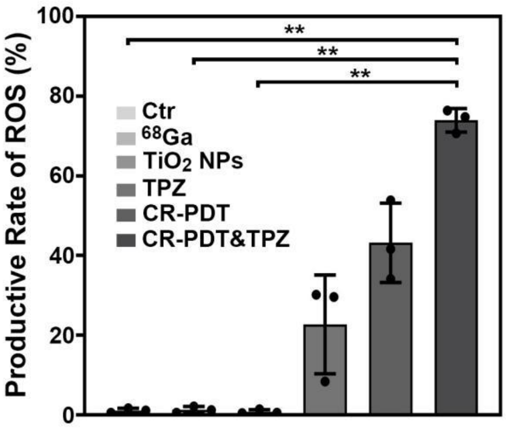

Figure S15. Quantitative comparison of the productive rata of ROS in cells of different groups (n = 3, mean ± s.d., $**P < 0.01$, one-way ANOVA with Dunnett's T3 test).

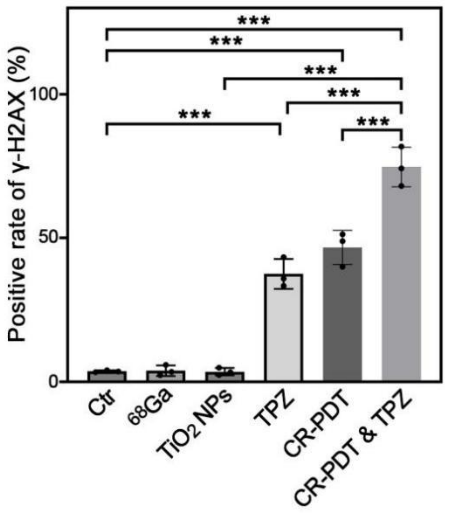

Figure S16. Quantitative comparison of fluorescence intensity of γ-H_2AX in cells of different groups (n = 3, mean ± s.d., $***P < 0.001$, one-way ANOVA with LSD-t test).

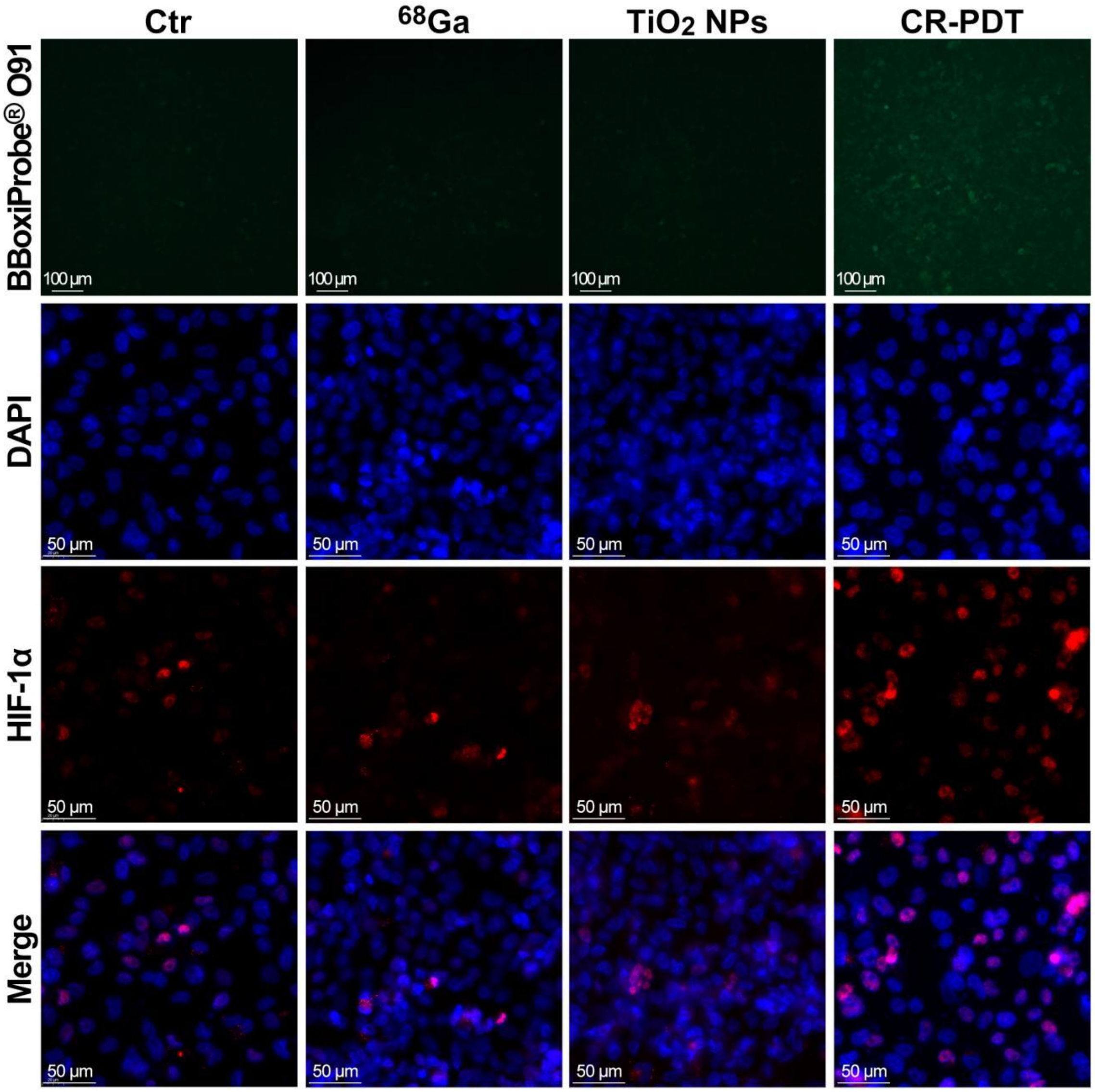

Figure S17. Cellular oxygen condition test by the BBoxiProbe® O91 and HIF-1α in cells treated by CR-PDT.

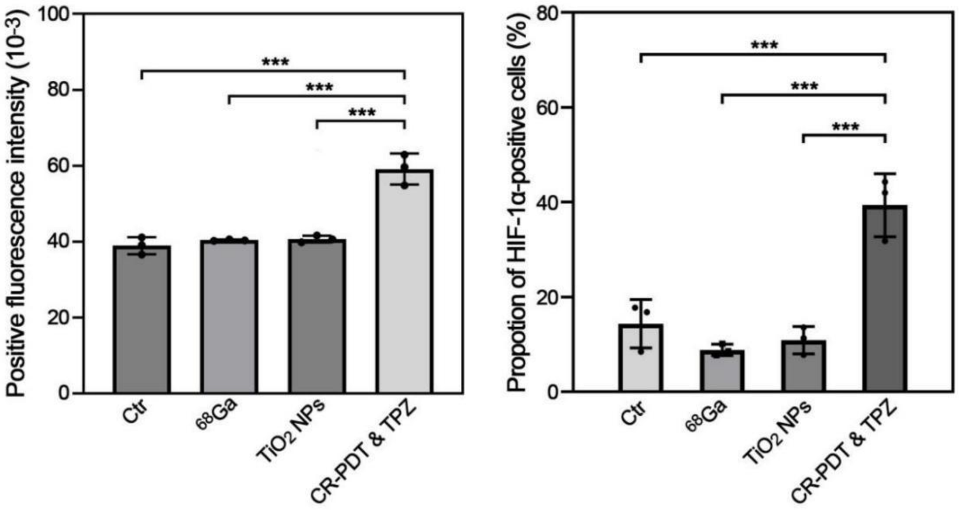

Figure S18. Quantitative analysis of BBoxiProbe® O91 (A) and HIF-1α (n = 5, mean ± s.d., ****P* < 0.001, one-way ANOVA with LSD-*t* test).

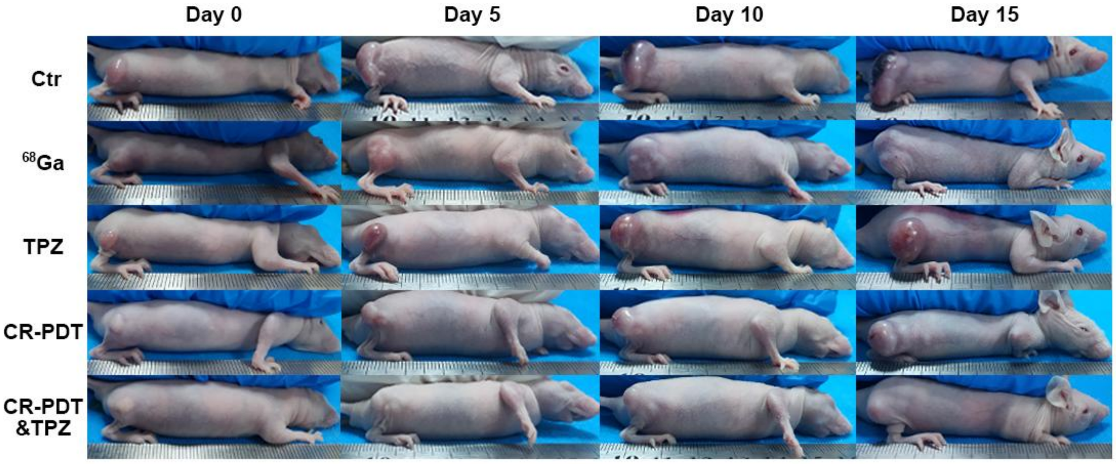

Figure S19. Typical digital photographs recording the growth process of tumor-bearing mice every five days from different treatments start.

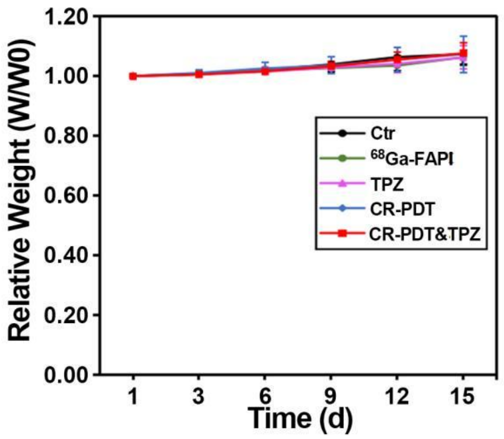

Figure S20. Time-course body weight (W) change of mice relative to the initial weight at 0 d P.I. (W_0) within 15 days after different treatments (n = 5, mean ± s.d).

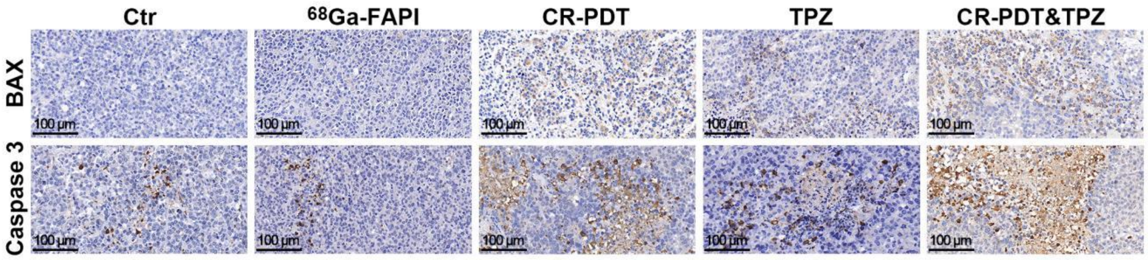

Figure S21. Representative immunohistochemistry images of pro-apoptotic proteins including Caspase 3 and BAX in tumor slices collected at day 15 after different treatments (original magnification: 400 ×).

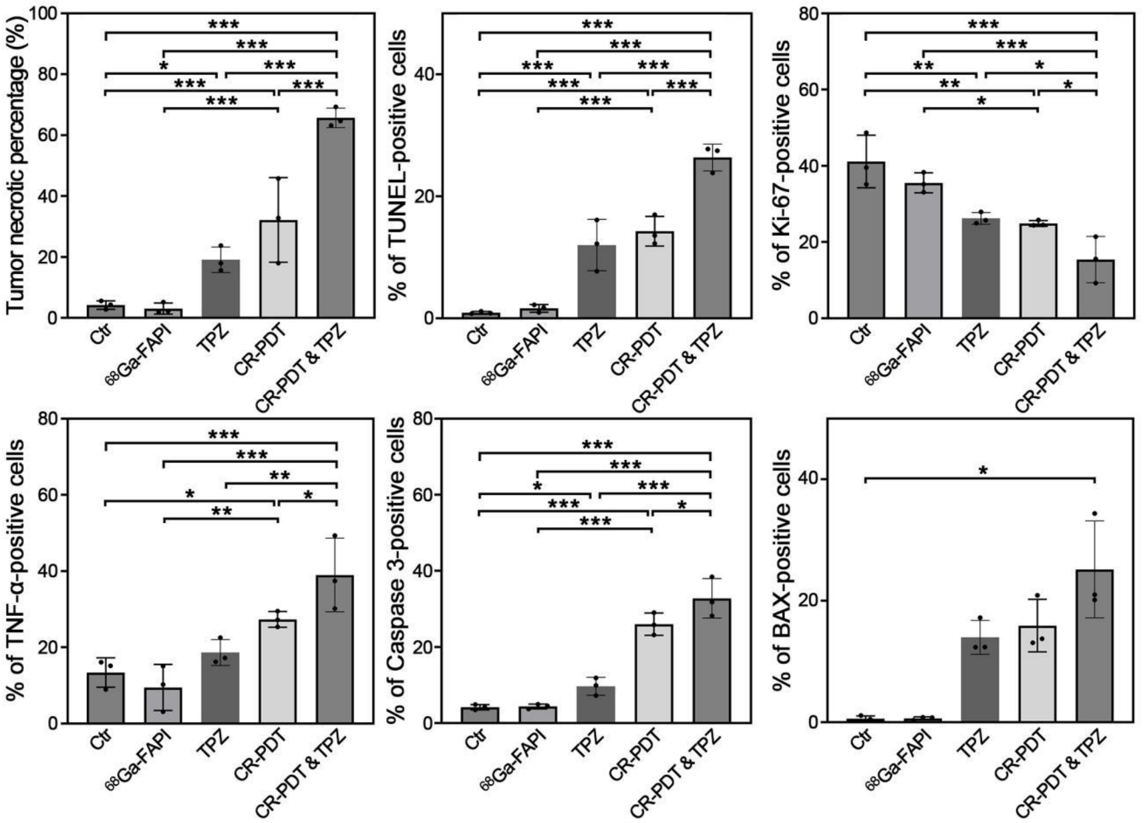

Figure S22. Quantitative analysis for H&E, TUNEL and immunohistochemistry images of Ki-67 and TNF-α in **Figure 5F**, as well as immunohistochemistry images of Caspase 3 and BAX in **Figure S21** (n = 3, mean ± s.d., $^{*}P < 0.05$, $^{**}P < 0.01$,$^{***}P < 0.001$, Kruskal-Wallis 1-way ANOVA for group comparison of BAX; one-way ANOVA with LSD-*t* test for group comparison of other indexes).

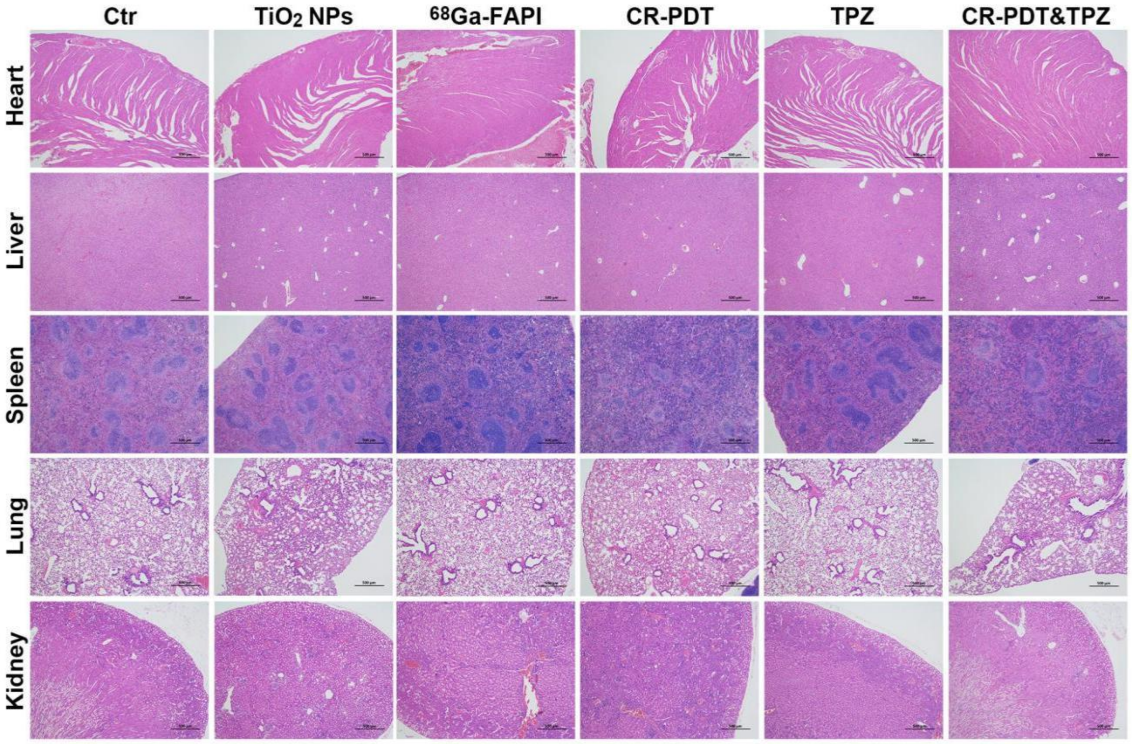

Figure S23. H&E staining for major organs of immunocompetent BALB/c mice to test the biosecurity of different treatments (original magnification: 40 ×).

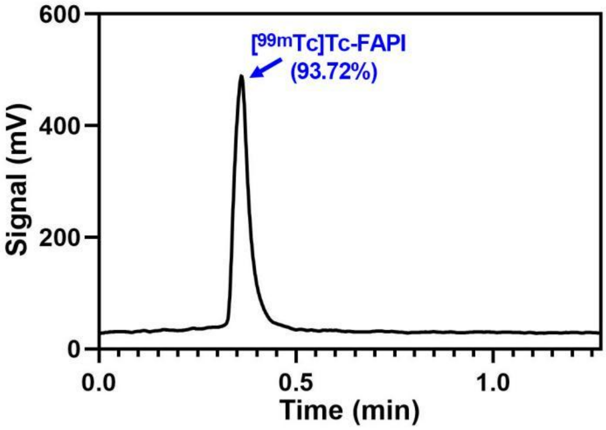

Figure S24. Radio thin-layer chromatography traces of [^{99m}Tc]Tc-FAPI.

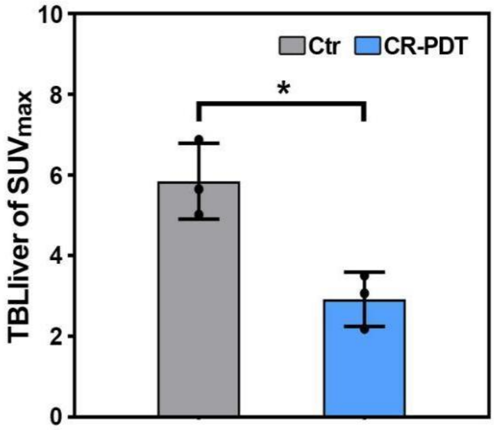

Figure S25. Semi-quantitative analysis of the TBRliver of SUVmax on [^{99m}Tc]Tc-FAPI SPECT/CT images of mice in CR-PDT and control groups (n = 3, mean ± s.d., **P* < 0.05, unpaired two-tailed *t*-test).

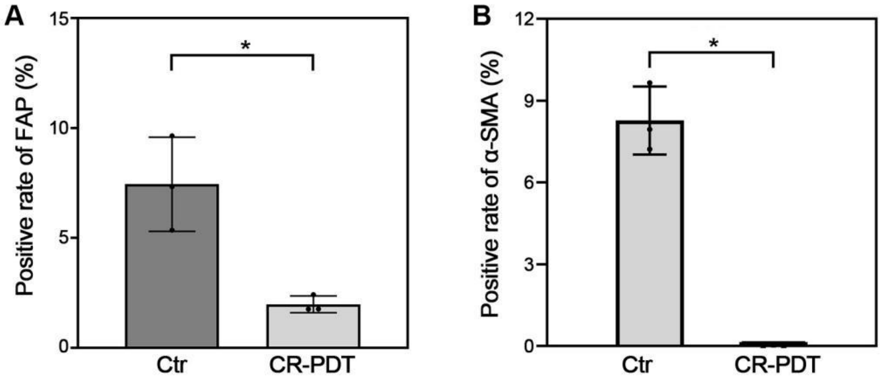

Figure S26. Quantitative analysis of FAP and α-SMA expression levels in tumors from the control and CR-PDT groups (n = 3, mean ± s.d., **P* < 0.05, Mann-Whitney U test).

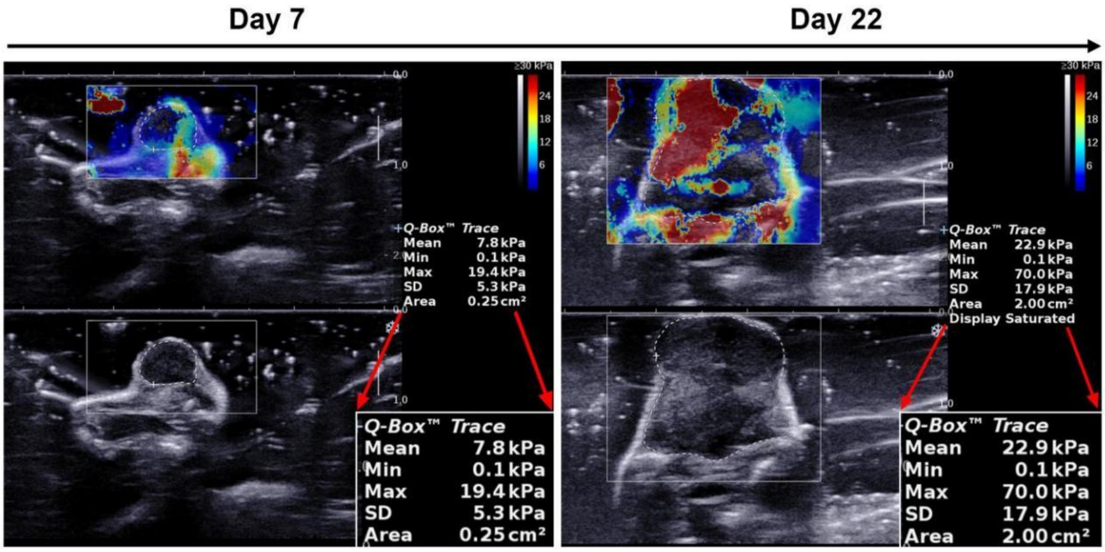

Figure S27. Ultrasonic elastography monitoring the change of tumor stiffness during the natural growth without any intervene. Tumors were imaged at day 7 and day 22 after the cancer cells were injected to construct tumor-bearing models.

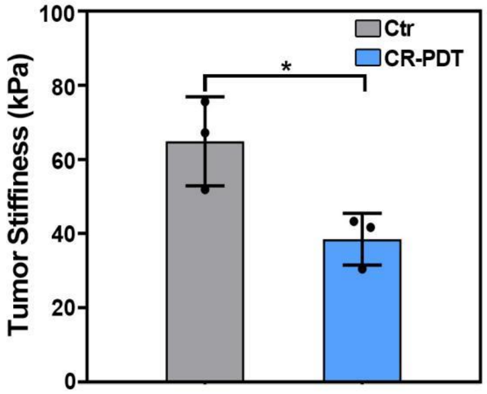

Figure S28. Semi-quantitative analysis based on ultrasonic elastography for comparing the stiffness of tumors treated by CR-PDT or not (n = 3, mean ± s.d., *P < 0.05, unpaired two-tailed t-test).

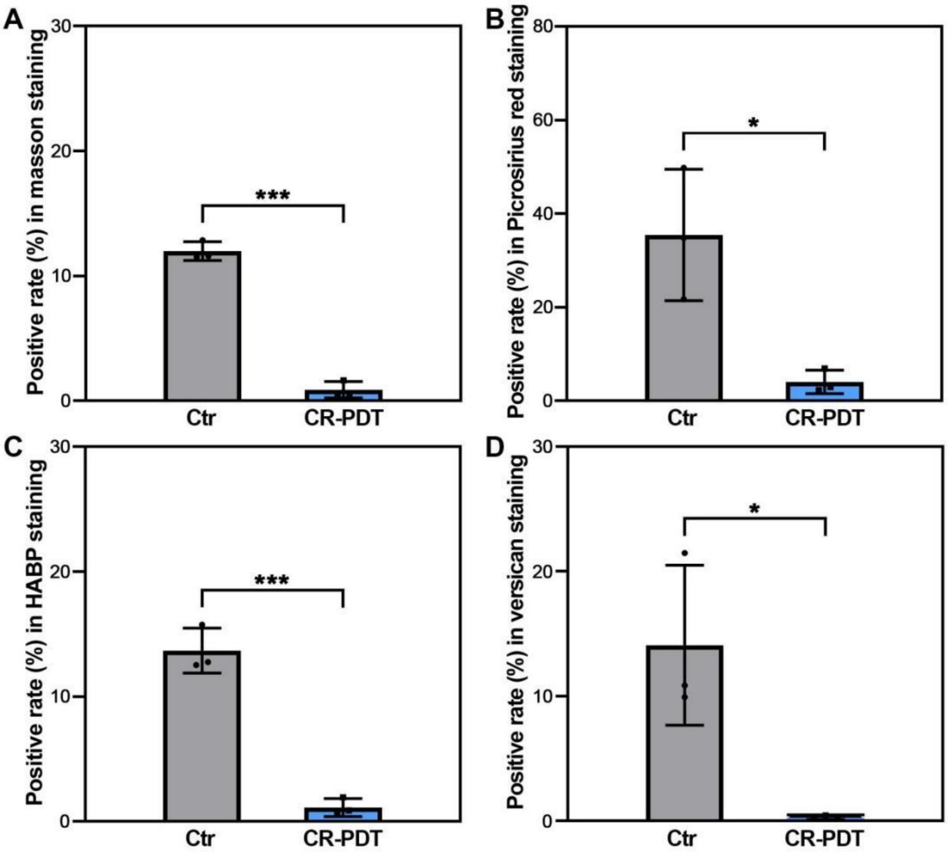

Figure S29. Quantitative analysis of tumor ECM. (AB) Infiltration extent of collagen fibers by Masson's trichrome staining and Picrosirius red staining. (CD) Deposition levels of HA and versican in tumors from the control and CR-PDT groups. (n = 3, mean ± s.d., $*P < 0.05$, $***P < 0.001$, Mann-Whitney U test for versican quantification, unpaired two-tailed *t*-test for others).

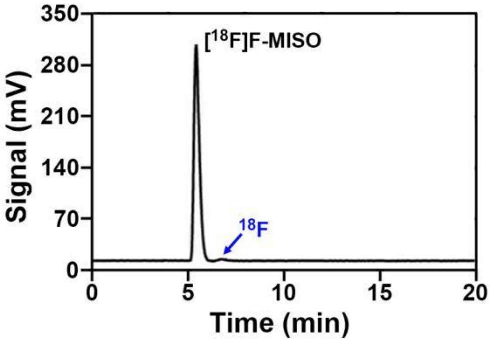

Figure S30. Radio labeling rate of $[^{18}F]$F-MISO based on HPLC.

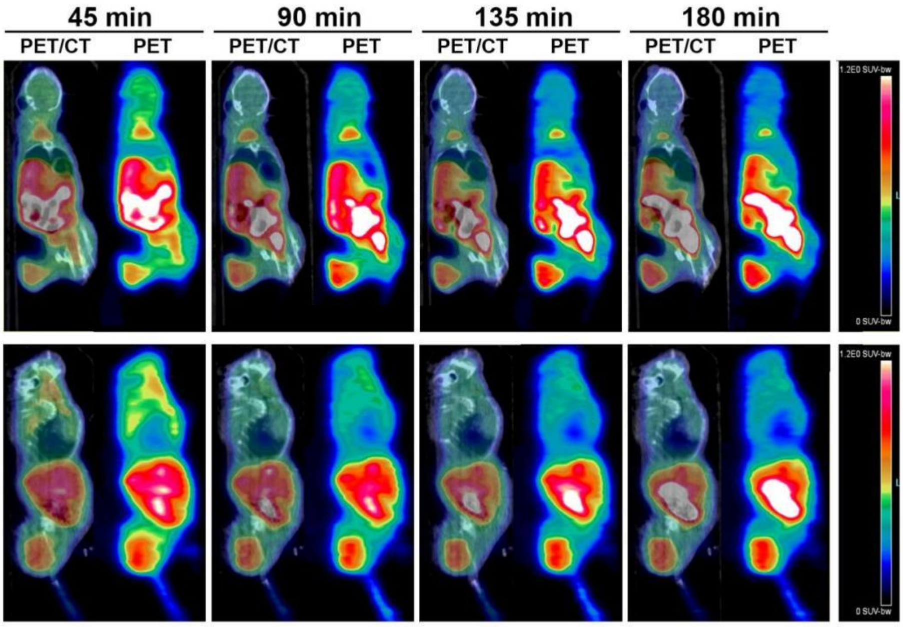

Figure S31. [18F]F-MISO PET/CT imaging of mice after intravenously injected with [18F]F-MISO for varying time.

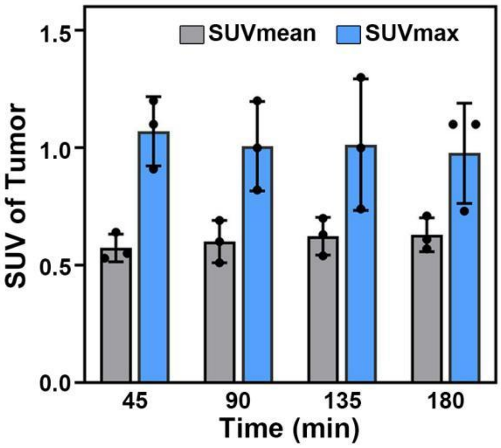

Figure S32. Quantitatively analyzing the change of SUVmax and SUVmean on [18F]F-MISO PET/CT imaging at different time points after injection.

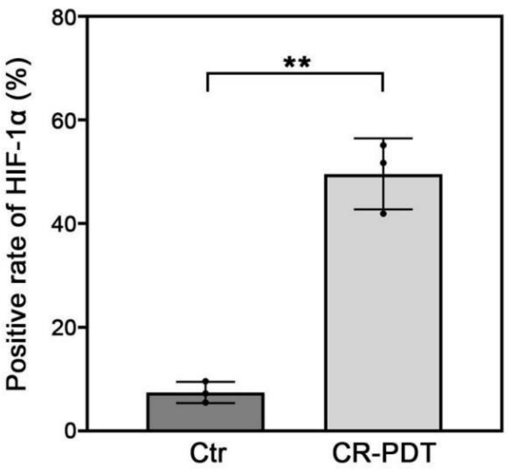

Figure S33. Quantitative analysis of HIF-1α expression in tumors from the control and CR-PDT groups (n = 3, mean ± s.d., $^{**}P < 0.01$, unpaired two-tailed *t*-test).

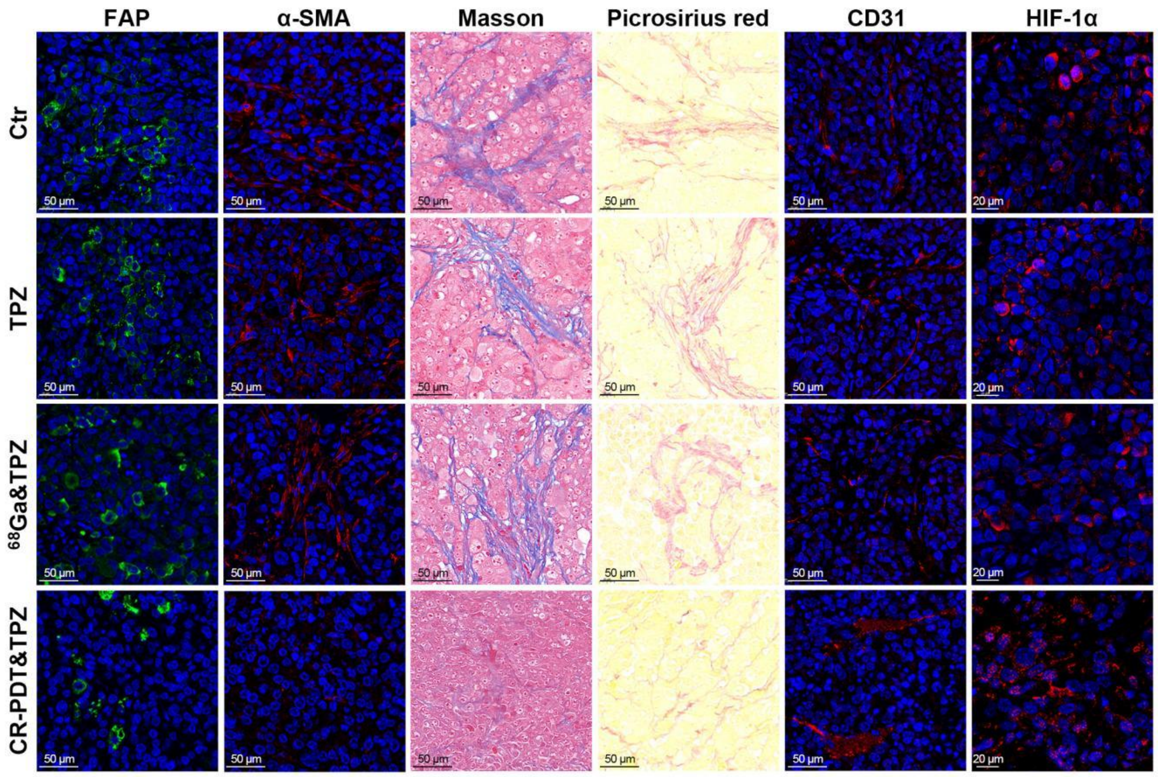

Figure S34. Evaluating the extracellular matrix components and hypoxic microenvironment in tumors treated by TPZ, [^{68}Ga]Ga&TPZ or CR-PDT&TPZ.

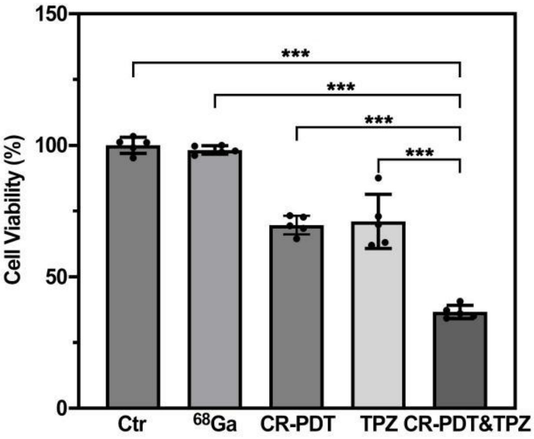

Figure S35. CCK-8 assay testing the killing efficiency of CR-PDT and TPZ on CAFs (n = 5, mean ± s.d., ***P < 0.001, one-way ANOVA with Dunnett's T3 test).

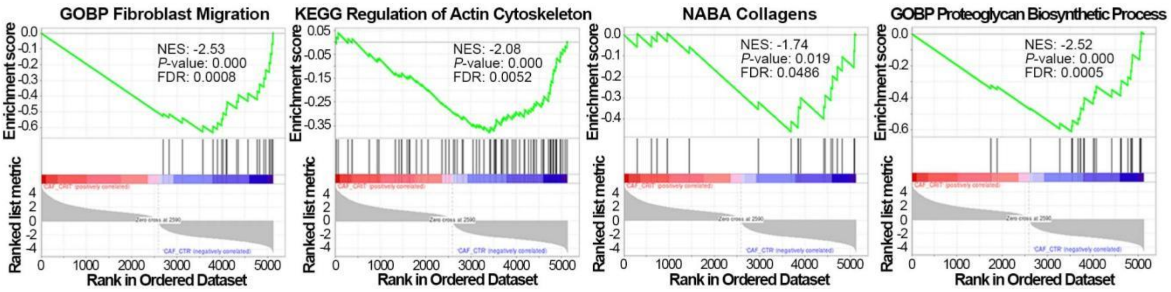

Figure S36. GSEA results showing downregulated gene sets in cells treated with CR-PDT.

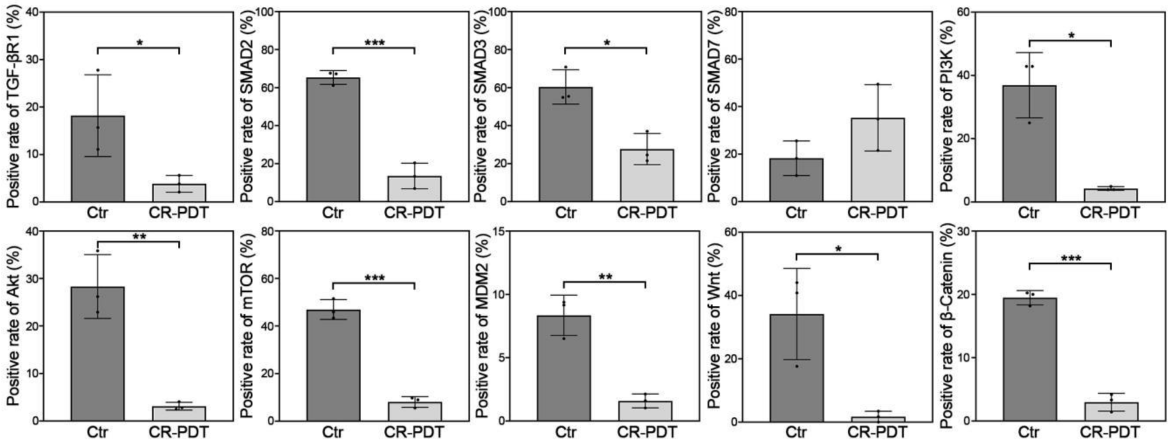

Figure S37. Quantitative analysis of representative regulators from the immunofluorescence staining results in **Figure 7M.** (n = 3, mean ± s.d., *P < 0.05, **P <

0.01, ***P < 0.001, Mann-Whitney U test for group comparison of PI3K; unpaired two-tailed t-test for group comparison of other regulators).

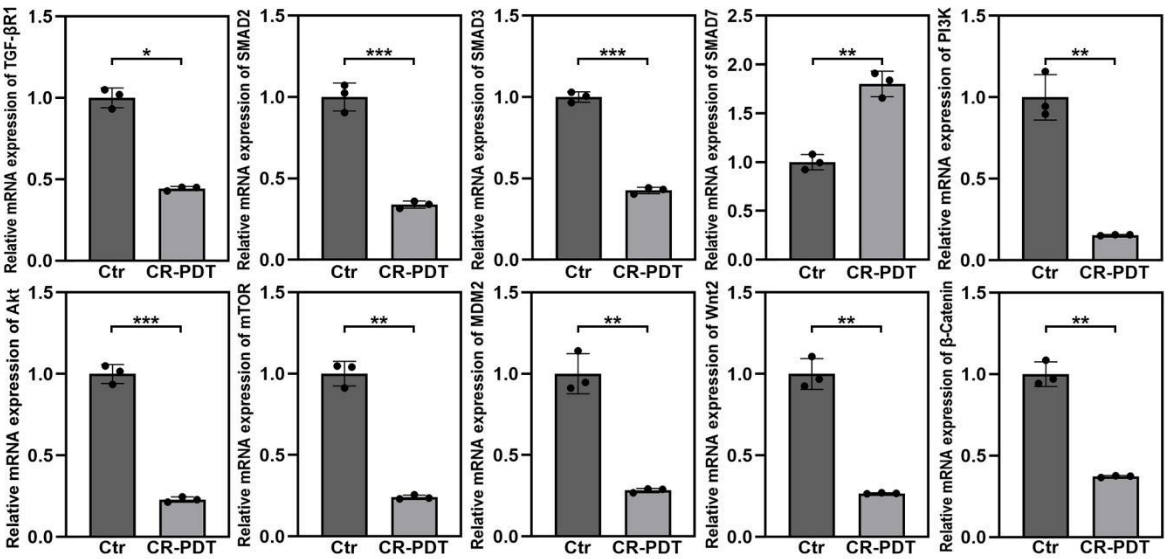

Figure S38. PCRs analyzing mRNA expression of representative regulators in RNA sequencing. (n = 3, mean ± s.d., *P < 0.05, **P < 0.01, ***P < 0.001, Mann-Whitney U test for group comparison of TGF-βR1; unpaired two-tailed t-test for group comparison of other regulators).

Group	Mean[a]				Median			
			95% Confidence Interval				95% Confidence Interval	
	Estimate	Std. Error	Lower Bound	Upper Bound	Estimate	Std. Error	Lower Bound	Upper Bound
Ctr	37.250	3.881	29.643	44.857	34.000	6.010	22.220	45.780
^{68}Ga	37.625	2.976	31.793	43.457	36.000	4.714	26.760	45.240
CR-PDT	51.875	2.923	46.146	57.604	50.000	4.714	40.760	59.240
TPZ	53.000	3.869	45.417	60.583				
CR-PDT&TPZ	58.375	1.520	55.396	61.354				
Overall	47.625	1.929	43.844	51.406	50.000	4.422	41.334	58.666

Table S1. The median or mean survival time of mice over the 60-day observation period post different treatments.

Group		Ctr		^{68}Ga		CR-PDT		TPZ		CR-PDT&TPZ	
		Square	Sig.	Square	Sig.	Square	Sig.	Square	Sig.	Square	Sig.
Log Rank (Mantel-Cox)	Ctr			.238	.626	6.997	.008	7.823	.005	14.368	.000
	^{68}Ga	.238	.626			7.392	.007	8.223	.004	14.817	.000
	CR-PDT	6.997	.008	7.392	.007			.678	.410	3.755	.053
	TPZ	7.823	.005	8.223	.004	.678	.410			1.317	.251
	CR-PDT&TPZ	14.368	.000	14.817	.000	3.755	.053	1.317	.251		

Table S2. Pairwise comparisons of the survival time distribution.
